# Senescence induction dictates response to chemo- and immunotherapy in preclinical models of ovarian cancer

**DOI:** 10.1073/pnas.2117754119

**Published:** 2022-01-26

**Authors:** Stella V. Paffenholz, Camilla Salvagno, Yu-Jui Ho, Matthew Limjoco, Timour Baslan, Sha Tian, Amanda Kulick, Elisa de Stanchina, John E. Wilkinson, Francisco M. Barriga, Dmitriy Zamarin, Juan R. Cubillos-Ruiz, Josef Leibold, Scott W. Lowe

**Affiliations:** ^a^Cancer Biology and Genetics Program, Sloan Kettering Institute, Memorial Sloan Kettering Cancer Center, New York, NY 10065;; ^b^Louis V. Gerstner Jr. Graduate School of Biomedical Sciences, Memorial Sloan Kettering Cancer Center, New York, NY 10065;; ^c^Department of Obstetrics and Gynecology, Weill Cornell Medicine, New York, NY 10065;; ^d^Sandra and Edward Meyer Cancer Center, Weill Cornell Medicine, New York, NY 10065;; ^e^Department of Molecular Pharmacology, Memorial Sloan Kettering Cancer Center, New York, NY 10065;; ^f^Department of Pathology, University of Michigan School of Medicine, Ann Arbor, MI 48109;; ^g^Department of Medicine, Gynecologic Medical Oncology Service, Memorial Sloan Kettering Cancer Center, New York, NY 10065;; ^h^Weill Cornell Graduate School of Biomedical Sciences, Cornell University, New York, NY 10065;; ^i^Department of Medical Oncology & Pneumology (Internal Medicine VIII), University Hospital Tuebingen, Tuebingen 72076, Germany;; ^j^DFG Cluster of Excellence 2180 “Image-Guided and Functional Instructed Tumor Therapy” (iFIT), University of Tuebingen, 72076 Tuebingen, Germany;; ^k^Howard Hughes Medical Institute (HHMI), Memorial Sloan Kettering Cancer Center, New York, NY 10065

**Keywords:** mouse models, ovarian cancer, cancer immunotherapy, senescence

## Abstract

High-grade serous ovarian carcinoma (HGSOC) is a cancer with dismal prognosis due to the limited effectiveness of existing chemo- and immunotherapies. To elucidate mechanisms mediating sensitivity or resistance to these therapies, we developed a fast and flexible autochthonous mouse model based on somatic introduction of HGSOC-associated genetic alterations into the ovary of immunocompetent mice using tissue electroporation. Tumors arising in these mice recapitulate the metastatic patterns and histological, molecular, and treatment response features of the human disease. By leveraging these models, we show that the ability to undergo senescence underlies the clinically observed increase in sensitivity of homologous recombination (HR)–deficient HGSOC tumors to platinum-based chemotherapy. Further, cGas/STING-mediated activation of a restricted senescence-associated secretory phenotype (SASP) was sufficient to induce immune infiltration and sensitize HR-deficient tumors to immune checkpoint blockade. In sum, our study identifies senescence propensity as a predictor of therapy response and defines a limited SASP profile that appears sufficient to confer added vulnerability to concurrent immunotherapy and, more broadly, provides a blueprint for the implementation of electroporation-based mouse models to reveal mechanisms of oncogenesis and therapy response in HGSOC.

## Significance

Efforts to understand and find new treatment options for high-grade serous ovarian cancer (HGSOC) have been confounded by a paucity of immune-competent models that accurately reflect the genetics and biology of the disease. Here, we leverage somatic tissue engineering to develop a fast and flexible immune-competent mouse model of HGSOC and reveal mechanistic insights into factors that dictate the response of ovarian tumors to conventional chemotherapy and immune checkpoint blockade. Our results identify a genotype-dependent therapy-induced senescence program that mediates sensitivity and resistance to first line chemotherapy and point to strategies to harness the senescence program to sensitize ovarian tumors to immune checkpoint blockade.

Over 70% of women diagnosed with high-grade serous ovarian carcinoma (HGSOC) succumb to their disease, making it the deadliest gynecological cancer (1). The standard of care for most patients consists of surgical debulking and platinum/taxane-based chemotherapy, though responses are typically transient, and resistance invariably emerges. Despite recent advances in targeted therapies such as poly (adenosine diphosphate [ADP]-ribose) polymerase (PARP) inhibitors and antiangiogenic therapies, survival has only marginally improved in the past 30 y (1). Moreover, immune checkpoint blockade (ICB), which has revolutionized the treatment of several cancer types (2–4), shows only modest results in HGSOC (5–7). Yet, little is known about molecular mechanisms that dictate response or resistance to any of these modalities.

HGSOC can be divided into specific subtypes that exhibit distinct clinical behaviors (8). The disease is characterized by an almost universal appearance of *TP53* mutations and an unusually high rate of copy number alterations (CNAs) (9) that target a range of known oncogenic events such as gains of the oncogene *MYC*. Moreover, tumors also harbor inactivating mutations in genes important for homologous recombination (HR) DNA repair, most commonly in *BRCA1* and *BRCA2* (9), which display an even greater degree of genomic rearrangements than HR-proficient tumors (10). HR deficiency sensitizes ovarian tumors to platinum-based therapies and PARP inhibitors (11) and, in other cancers, appears to sensitize tumors to immune-modulating agents, but it is unclear to what extent this process plays a role in HGSOC (12, 13). Clearly, a better understanding of the biological and molecular mechanisms responsible for genotype-response patterns would enable existing therapies to be used more effectively and facilitate development of novel strategies to overcome resistance.

Relating clinical observations to mechanisms requires the availability of accurate model systems. However, until recently, models that faithfully recapitulate the heterogeneity of human HGSOC have been limited. Genetically engineered mouse models (GEMMs), which are generated by intercrossing a series of tissue-specific and/or conditional alleles and result in production of autochthonous tumors, have helped elucidate the consequences of cancer-associated mutations on HGSOC tumorigenesis (14). While such autochthonous models are powerful, they are time consuming, expensive, and the specific requirement for female mice leads to substantial animal waste. Consequently, it is impractical to develop animal cohorts of sufficient size and genotypic diversity for rapid and rigorous mechanistic and preclinical studies. Recently, both patient and murine HGSOC organoid models covering a range of genomic configurations have been developed, which enable perturbations in vitro or following orthotopic transplantation in vivo (15–19). However, these systems also have limitations: the human models cannot be studied in the presence of the intact immune system and the murine models that employ in vitro transformed cells do not undergo immunoediting and lack other microenvironmental factors that shape tumor development in vivo (20, 21).

Considering the need for more accurate and facile autochthonous models, we combined CRISPR genome engineering approaches with transposon/transposase-based systems and in vivo organ electroporation (EPO-GEMM) to model HGSOC in mice. The EPO-GEMM approach allows the study of autochthonous tumors in an immune-competent background while overcoming the logistical disadvantages of traditional GEMMs. Using this approach, we developed genetically and histopathologically accurate models of HGSOC and use them to gain mechanistic insights into genotype-dependent therapy responses to chemo- and immunotherapies.

## Results

### Somatic Introduction of Oncogenic Lesions Generates High-Grade Serous Ovarian Carcinoma.

**Fig. 1.**
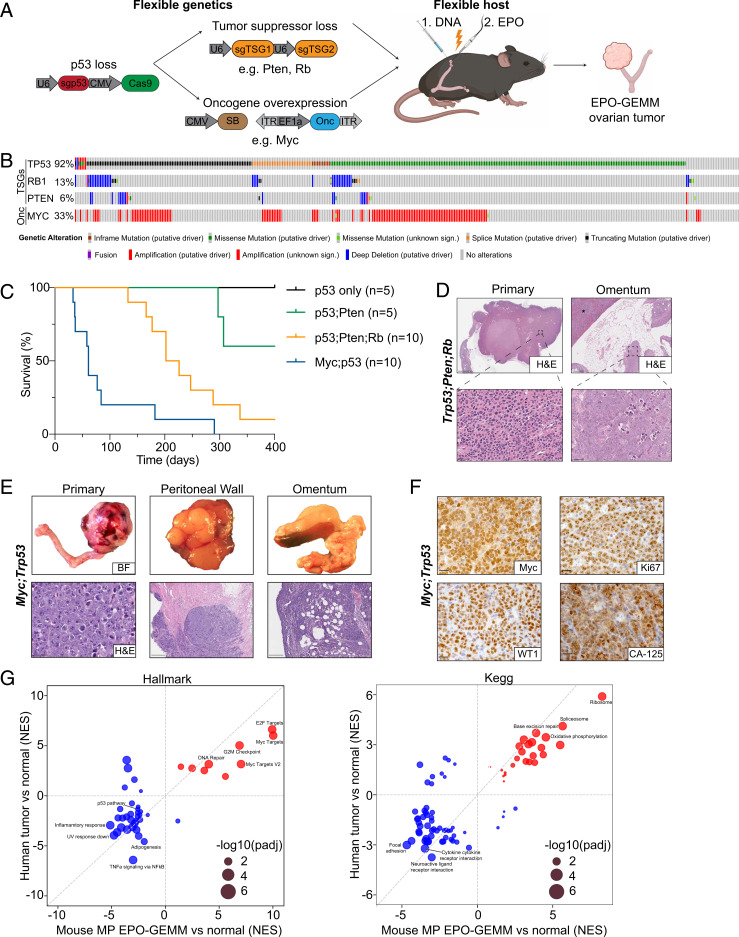
Somatic introduction of oncogenic lesions generates high-grade serous ovarian carcinoma. (*A*) Schematic of the EPO-GEMM approach to generate ovarian cancer. A CRISPR-Cas9 vector targeting *Trp53* is codelivered with additional sgRNAs targeting tumor suppressor genes (TSGs) or an oncogene (Onc) containing transposon vector in combination with a Sleeping Beauty (SB) transposase into the ovary and fallopian tube by direct in vivo electroporation. (*B*) Oncoprint displaying the genomic status of *TP53*, *RB1*, *PTEN*, and *MYC* in HGSOC (The Cancer Genome Atlas [TCGA], Pan-cancer dataset). (*C*) Kaplan–Meier survival curve of C57BL/6 mice electroporated with the indicated combinations of plasmids. (*D*) Representative hematoxylin and eosin (H&E) staining of a Trp53;Pten;Rb EPO-GEMM primary tumor (*Left*) and omentum with a micrometastasis (*Right*). (Scale bar, 5 mm [*Top Left*], 200 µm [*Top Right*], 50 µm [*Bottom Left*], 25 µm [*Bottom Right*].) The spleen is labeled with an asterisk. (*E*) Macroscopic bright-field (BF) images (*Top*) and H&E sections (*Bottom*) of genital tracts, peritoneum, and omentum of a mouse bearing a MP EPO-GEMM tumor. (Scale bar, 50 µm [*Bottom Left*], 500 µm [*Bottom Middle* and *Right*].) (*F*) Representative immunohistochemical staining of a MP EPO-GEMM ovarian tumor for MYC, the proliferation marker Ki67, and the HGSOC markers Wilms Tumor 1 (WT1) and Cancer Antigen 125 (CA-125) in representative sections. (Scale bar, 20 µm.) (*G*) Correlation of gene set enrichment analysis normalized enrichment scores (NES) derived from RNA-seq data for Hallmark (*Left*) or KEGG (*Right*) pathways enriched in human ovarian cancer samples (*y* axis) and murine EPO-GEMM ovarian cancer (*x* axis) compared to normal tissue. Highlighted are key pathways; circle size represents the adjusted *P* value.

To develop murine genotypically diverse models of HGSOC, we optimized methods to introduce genetic elements into the ovary by direct tissue electroporation. Briefly, the ovary is surgically exposed and injected with plasmid DNA encoding CRISPR-Cas9 constructs and/or a transposon vector and a Sleeping Beauty transposase, followed by electroporation of the surrounding ovarian and fallopian tube tissue (Fig. 1*A*). Since more than 95% of HGSOC patients harbor tumors with mutations in the *TP53* tumor suppressor gene (9), all genotypic configurations included vectors coexpressing Cas9 and a single-guide RNA (sgRNA) targeting *Trp53* that was previously validated in vivo (22). In addition, various combinations of oncogene-expressing transposon vectors or sgRNAs targeting additional tumor suppressor genes that co-occur in human patients were included (Fig. 1*B*). Following electroporation, mice were monitored for tumor onset and progression by ultrasound imaging and abdominal palpation. To determine the extent to which our model recapitulates human HGSOC, murine tumor material was analyzed histologically for clinically relevant HGSOC biomarkers and molecularly for CRISPR-Cas9–engineered somatic mutations, acquired CNAs, and transcriptional profiles.

Disruption of *Trp53* alone did not produce tumors over the time course of these experiments. Focal tumors arose following electroporation of sgRNAs targeting *Trp53* and *Pten*, or *Trp53*, *Pten*, and *Rb1* with 40% and 90% penetrance, respectively, with the latter configuration displaying a median survival of 214 d. Traditional GEMMs with analogous genetic configurations develop tumors with similar penetrance and latency (23–26). Since *MYC* gain or amplification often co-occurs with *TP53* mutations in human HGSOC and can be oncogenic in transplantation models (27, 28), we also combined *Trp53* sgRNAs together with a transposon vector overexpressing *MYC*. The combination of *MYC* overexpression with CRISPR-Cas9–induced loss of *Trp53* was particularly potent to produce tumors in 100% of the recipients and vastly accelerate the disease (Fig. 1*C*, median survival 61 d).

Most mice developed metastatic disease to the omentum and peritoneum, which are the most common sites of metastatic spread in patients (29). While tumors arising through the disruption of tumor suppressors only generated micrometastatic nodules in the omentum (Fig. 1*D*), the addition of *MYC* resulted in macrometastatic disease and ascites formation (Fig. 1*E*). As occurs in human HGSOC tumors (30), EPO-GEMM tumors exhibited a solid architecture with some glandular areas, necrosis in solid areas, large hyperchromatic nuclei, and abundant, often atypical, mitotic figures (Fig. 1 *D* and *E*). Histologically, we never observed sarcomas or lymphomas arising in electroporated animals and, accordingly, all tumors analyzed expressed molecular hallmarks of human HGSOC, including Cytokeratin-7 (CK7), Wilms Tumor 1 (WT1), Cancer Antigen 125 (CA-125), Paired box 8 (Pax-8), and high Ki67 (Fig. 1*F* and *SI Appendix*, Fig. S1*A*). These markers were retained in metastases (*SI Appendix*, Fig. S1*B*), with the exception of CK7 whose reduced expression at metastatic sites correlates with poor prognosis in patients (31). As expected, *MYC*-driven tumors showed high levels of MYC protein expression (Fig. 1*F*).

At the molecular level, Sanger sequencing analysis of the resulting tumors at terminal stage confirmed the presence of insertion and deletion mutations (indels) at the *Trp53*, *Pten*, and *Rb1* loci, consistent with their disruption through CRISPR-Cas9 (*SI Appendix*, Fig. S1*C*). Deep sequencing of the CRISPR-Cas9–induced *Trp53* scar revealed that tumors were oligoclonal and that the dominant clones were shared between ovarian tumors and paired omentum metastases, confirming that the disseminated cells arose from the primary tumor site (*SI Appendix*, Fig. S1*D*). Analysis of CNAs using sparse whole-genome sequencing (32) of primary EPO-GEMM tumors revealed widespread aneuploidies as occurs in human disease (33). Recurrent changes included loss of mouse chromosomes 10 and 12 and gains of chromosomes 1 and 2. As occurs in a traditional GEMM with *Brca1;Trp53;Rb1;Nf1* genotype (23), some tumors generated by tumor suppressor gene inactivation without *MYC* overexpression showed gain or amplification of the *Myc* locus on mouse chromosome 15 (*SI Appendix*, Fig. S1*E*) together with MYC protein expression (*SI Appendix*, Fig. S1*F*). These data underscore the importance of *MYC* in driving HGSOC and support the rationale for using *MYC* as a driver in our EPO-GEMM platform.

Analysis of RNA-sequencing (RNA-seq) data derived from tumors demonstrated that the *MYC;Trp53* (MP) EPO-GEMM system faithfully recapitulates the transcriptional states characteristic of human disease (Fig. 1*G*). When compared to normal tissue, the top up-regulated pathways in both the Hallmark and Kyoto Encyclopedia of Genes and Genomes (KEGG) databases were related to proliferation (Hallmark: E2F targets, MYC targets, and G2M checkpoint; KEGG: ribosome, spliceosome, and oxidative phosphorylation) and DNA repair (Hallmark: DNA repair; KEGG: base excision repair) and the top down-regulated pathways were related to an active immune response (Hallmark: TNFa signaling via NF-κB and inflammatory response; KEGG: neuroactive ligand receptor interaction and cytokine receptor interaction). In line with the almost ubiquitous *TP53* inactivation in HGSOC, the p53 pathway was among the top down-regulated pathways in the Hallmark gene sets. These results validate the EPO-GEMM approach as a flexible platform to model HGSOC tumors of varying genotypes that resemble the metastatic, histological, genomic, and transcriptomic properties of the human disease.

The similarity of EPO-GEMM ovarian cancers to human HGSOC was striking, given that our electroporation method does not discriminate between cell types within the targeted tissue. To confirm that the tumors originated from epithelial cells, we harnessed the flexibility of the EPO-GEMM approach to directly mutagenize Cytokeratin-8 (CK8)-expressing epithelial cells, a cell type that can serve as a tumor-initiating cell in the absence of *Trp53* (34), and is also retained in traditional GEMMs arising in *Brca1;Trp53;Pten*-deficient mice (24). Double transgenic mice harboring a CRE-estrogen receptor fusion transgene (CreER) under the control of the CK8 promoter and a Lox-Stop-Lox (LSL) Cas9-IRES-GFP transgene were treated with tamoxifen and electroporated with vectors expressing a *MYC* transposon, a transposase, and a *Trp53* sgRNA. In this setting, only CK8-positive epithelial cells are capable of CRISPR-Cas9–mediated editing upon tamoxifen addition (*SI Appendix*, Fig. S1*G*). Tamoxifen treatment triggered expression of GFP in CK8-positive epithelial cells (*SI Appendix*, Fig. S1*H*) and led to the formation of GFP-positive ovarian tumors with similar histological and transcriptional features as observed in wild-type (WT) mice harboring tumors of the same genotype (*SI Appendix*, Fig. S1 *I*–*K*). These data confirm the epithelial origin of the EPO-GEMM tumors and imply that relevant epithelial populations in the ovary are most sensitive to the genetic alterations that co-occur in the human disease.

### HR-Deficient Tumors Have Unique Genomic, Immune, and Therapy Response Features.

**Fig. 2.**
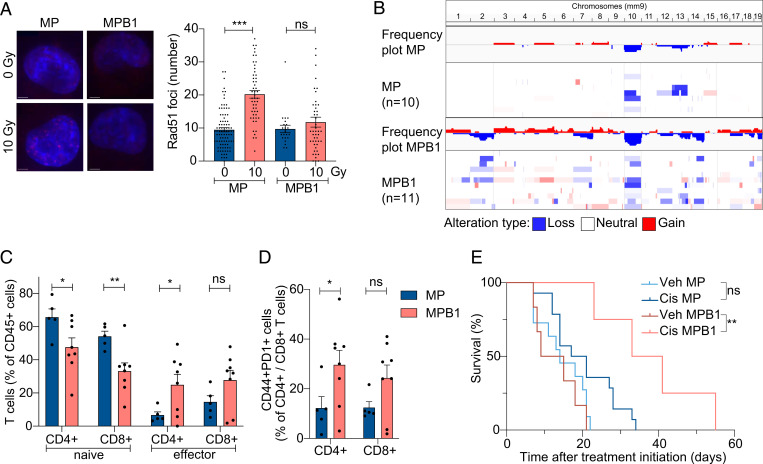
HR-deficient tumors have unique genomic, immune, and therapy response features. (*A*) Representative images showing IF staining of RAD51 (*Left*) and the quantification of the number of RAD51 foci per nuclei (*Right*). Cell nuclei were stained with DAPI (blue). (Scale bar, 5 µm.) (*B*) Frequency plot of CNA analysis of MP (*n* = 10) and MPB1 (*n* = 11) EPO-GEMM ovarian tumors. (*C* and *D*) Immune cell analysis by flow cytometric analysis of representative EPO-GEMM tumors of the indicated genotypes (*n* = 5 to 8 mice per group). (*E*) Kaplan–Meier survival curve of C57BL/6 mice electroporated with the indicated combinations of plasmids and treated with vehicle (veh) or cisplatin (cis). Treatment was initiated after tumors were palpable (*n* = 4 to 13 mice per group). *P ≤ 0.05, **P ≤ 0.01, ***P ≤ 0.001, ns: not significant; mean ± SEM; analyses performed using unpaired *t* test (*A*, *C*, and *D*) and log-rank test (*E*).

More than one-third of ovarian cancer patients are classified as HR deficient (9). To model tumors arising in this important tumor subtype, we incorporated sgRNAs targeting *Brca1* into the MP combination described above using a vector that coexpresses *Trp53* and *Brca1* sgRNAs (*MYC;Trp53;Brca1*, MPB1) (*SI Appendix*, Fig. S2*A*). The resulting tumors displayed an onset and histology that was similar to those harboring *MYC* and *Trp53* alterations alone (*SI Appendix*, Fig. S2 *B* and *C*). Despite similar latencies, tumors produced with a plasmid mixture including the *Brca1* sgRNA invariably displayed indels at the *Brca1* sgRNA target site (*SI Appendix*, Fig. S2*D*), implying that *Brca1* inactivation produced a selective advantage during tumorigenesis. Accordingly, as is characteristic of HR-deficient cells (18), MPB1 tumor cells isolated from EPO-GEMM tumors showed reduced induction of Rad51-containing nuclear foci following irradiation compared to MP tumor cells with intact *Brca1* (Fig. 2*A*).

Compared to HR-proficient tumors, HR-deficient human ovarian cancers acquire even more genomic rearrangements (10), display substantial T cell infiltration (35–37), and are more responsive to platinum-based chemotherapy (38, 39). Similarly, murine MPB1 ovarian EPO-GEMM tumors harbored more CNAs (Fig. 2*B*) and a greater proportion of CD4^+^ and CD8^+^ T cells expressing both activation and exhaustion markers relative to *Brca1*-proficient counterparts (Fig. 2 *C* and *D* and *SI Appendix*, Fig. S2*E*). Furthermore, mice harboring primary MPB1 tumors showed significantly improved survival following cisplatin therapy (Fig. 2*E*), a result that was recapitulated in mice following subcutaneous or intraperitoneal (i.p.) injection of primary EPO-GEMM tumor-derived cell lines (*SI Appendix*, Fig. S2 *F*–*H*). Therefore, MPB1 EPO-GEMM tumors recapitulate key biological and clinical features of human HR-deficient tumors.

### Cisplatin Treatment Preferentially Induces Tumor Cell Senescence and Alters Immune Infiltrates in HR-Deficient HGSOC.

**Fig. 3.**
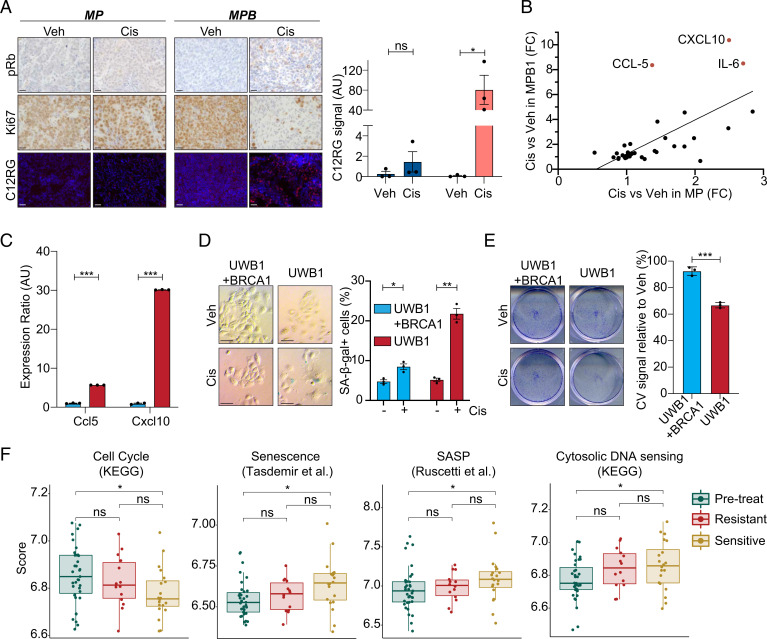
Cisplatin treatment preferentially induces tumor cell senescence and alters immune infiltrates in HR-deficient HGSOC. (*A*) Immunohistochemical staining of phospho-Rb (pRb) and Ki67 and staining of C12RG, a fluorogenic substrate for SA-β-gal activity, of subcutaneously transplanted tumors treated with vehicle or cisplatin. (Scale bar, 20 µm.) Quantification of SA-β-gal activity is shown on the *Right* (*n* = 3). (*B*) Cytokine expression in MP (*x* axis) or MPB1 (*y* axis) cell lines treated with cisplatin relative to vehicle (*n* = 2 independent cell lines per genotype). (*C*) RT-qPCR analysis of *Ccl5* and *Cxcl10* in cisplatin-treated *BRCA1*-proficient (UWB1 + BRCA1) or -deficient (UWB1) human ovarian cancer cells (*n* = 3 technical replicates). (*D*) SA-β-gal staining (*Left*) and quantification (*Right*) of either *BRCA1*-proficient (UWB1 + BRCA1) or -deficient (UWB1) human ovarian cancer cell lines after treatment with vehicle or 100 nM cisplatin for 6 d (*n* = 3). (Scale bar, 50 µm.) (*E*) Clonogenic crystal violet (CV) assay of human ovarian cancer cells replated in the absence of drugs after 6-d pretreatment as in *D* (*n* = 3). (*F*) Expression of senescence and SASP signatures in patient samples isolated pretreatment and after three cycles of chemotherapy during the Cambridge Translational Cancer Research Ovarian Study 01 (CTCR-OV01) clinical trial (49, 50) (GSE15622). Posttreatment samples are subdivided into resistant and sensitive cases. *P ≤ 0.05, **P ≤ 0.01, ***P ≤ 0.001, ns: not significant; mean ± SEM; analyses performed using unpaired *t* test (*A* and *C*–*E*) and Wilcoxon signed-rank test (*F*).

As a first step toward assessing mechanisms leading to genotypic differences in intrinsic cisplatin sensitivity, we analyzed the biological responses to cisplatin treatment. Cultured cells established from MP and MPB1 tumors showed similar levels of growth inhibition and apoptosis induction following cisplatin treatment in vitro (*SI Appendix*, Fig. S3 *A* and *B*) and in vivo (*SI Appendix*, Fig. S3*C*). In contrast, *Brca1*-deficient cells showed a much greater proclivity for senescence, displaying an increase in senescence-associated β-galactosidase (SA-β-gal) activity and a decrease in colony-forming potential following cisplatin treatment compared to the *Brca1*-proficient MP counterparts (*SI Appendix*, Fig. S3 *D* and *E*). Similar results were observed in vivo, with MPB1-derived tumors showing more SA-β-gal activity as measured by the fluorogenic substrate C12RG (40), reduced phosphorylated Rb, and a concomitant decrease in Ki67 staining compared to MP controls after short-term cisplatin treatment (Fig. 3*A*). Apparently, *Brca1* mutations sensitize ovarian tumor cells to cisplatin-induced senescence.

Senescence is a potent tumor suppressive mechanism that involves a stable proliferative arrest coupled to a secretory program known as the senescence-associated secretory phenotype (SASP) (41). The SASP alters the tumor microenvironment (TME), where it can modulate extracellular matrix, tumor vasculature, and the functionality of immune cells (42, 43) that, in some instances, produces an inflammatory TME (44, 45). To examine SASP in our system, we performed cytokine array analysis on a series of cisplatin-treated MP and MPB1 cell lines. Out of the 44 factors assessed in this panel, *Ccl5*, *Cxcl10*, and *Il6* were the most significantly increased in the *Brca1*-deficient cells (Fig. 3*B*) and this correlated with increased mRNA expression in several MPB1 cell lines (*SI Appendix*, Fig. S3*F*) and in an isogenic setting in which *Brca1* was disrupted in MP tumor cells following in vitro establishment (*SI Appendix*, Fig. S3*G*). Taxol, another frontline chemotherapy used for HGSOC treatment, also induced a similar cytokine profile in MPB1 tumor cells (*SI Appendix*, Fig. S3*H*). Interestingly, the SASP profile detected in MPB1 cells following chemotherapy treatment was more restricted than has been observed in other contexts (44, 45), showing predominant secretion of immune modulatory cytokines and no endothelial cell regulatory factors. Accordingly, we did not detect obvious changes in tumor vasculature as assessed by CD31 immunofluorescence (IF) following cisplatin treatment (*SI Appendix*, Fig. S3*I*).

We also examined cisplatin responses in human cancer cells and patients. We analyzed the *BRCA1*-mutant UWB1.289 cell line along with its isogenic counterpart with forced expression of the *BRCA1* WT gene (46). Cisplatin treatment led to induction of *CCL-5* and *CXCL-10* in the *BRCA1*-mutant cells, which was dampened by forced expression of *BRCA1*-WT (Fig. 3*C*). The *BRCA1*-mutant cells showed a more pronounced senescence response, as evaluated by increased SA-β-gal activity and a decreased colony-forming potential following cisplatin treatment (Fig. 3 *D* and *E*). Additionally, a well-characterized *BRCA1*-deficient breast cancer cell line (47) was also more prone to cisplatin-induced senescence than a *BRCA1*-proficient counterpart (*SI Appendix*, Fig. S3 *J* and *K*). In patients, a retrospective analysis of RNA-seq data from matched pre- and posttreatment samples (48) showed an enrichment for gene signatures linked to senescence and SASP posttherapy (*SI Appendix*, Fig. S3*L*) with *CCL-5* and *IL-6* being among the most enriched genes in these signatures. Furthermore, in a dataset where outcomes were known (49, 50) (GSE15622), expression of senescence signatures was higher in the sensitive tumors (Fig. 3*F*). We also observed higher expression of transcripts linked to the cytosolic DNA-sensing pathway in sensitive tumors, which has previously been associated with senescence (51). While this dataset did not allow for classification of patients based on BRCA or HR status, it is consistent with the notion that senescence induction improves outcomes in HGSOC patients. Thus, *Brca1* loss is sufficient to predispose ovarian cancer cells to induction of a chemotherapy-induced senescence program that appears associated with improved outcomes in patients.

### Cisplatin Treatment Leads to a cGas/STING-Dependent Infiltration of T and Natural Killer (NK) Cells in HR-Deficient Tumors.

**Fig. 4.**
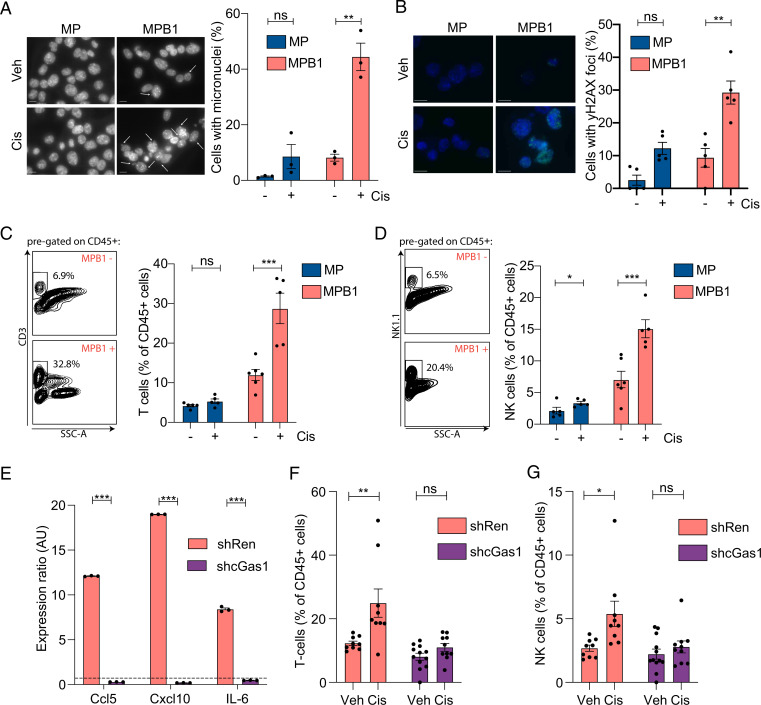
Cisplatin treatment leads to a cGas/STING-dependent infiltration of T and NK cells in HR-deficient tumors. (*A*) Micronuclei staining of MP or MPB1 cell lines treated with cisplatin. Cell nuclei were stained with DAPI (gray) (*n* = 3 independent cell lines per genotype). (Scale bar, 20 µm.) (*B*) γH2AX staining (green) of MP or MPB1 cell lines treated with cisplatin. Cell nuclei were stained with DAPI (blue) (*n* = 5). (Scale bar, 20 µm.) (*C* and *D*) Representative flow cytometry plots (*Left*) and quantification (*Right*) of T cell (*C*) or NK cell (*D*) infiltration in subcutaneously transplanted ovarian tumors after treatment with two cycles of cisplatin (*n* = 5 to 6 mice per group). (*E*) RT-qPCR analysis of *Ccl5*, *Cxcl10*, and *Il6* in cell lines containing control Renilla (shRen) or cGas shRNAs (shcGas). Expression ratio of cisplatin treated relative to vehicle treated is shown (*n* = 3). (*F* and *G*) Flow cytometry analysis of T (*F*) and NK (*G*) cell infiltration in subcutaneously transplanted MPB1 ovarian tumors containing control Renilla or cGas shRNAs after treatment with two cycles of cisplatin (*n* = 8 to 12 mice per group). *P ≤ 0.05, **P ≤ 0.01, ***P ≤ 0.001, ns: not significant; mean ± SEM; analyses performed using unpaired *t* test (*A*–*G*).

*Ccl5*, *Cxcl10*, and *Il6* are immune modulatory cytokines and members of the Interferon Stimulated Genes (ISG) family that act downstream of cGAS/STING signaling (52) and are often associated with the SASP (53, 54). cGAS is an intracellular innate immune sensor of cytosolic double-stranded DNA (55) that can be activated by nucleic acids present in micronuclei and can increase in cells with rampant genome instability (56). Breast and ovarian cancers harboring *BRCA* mutations have been shown to harbor high levels of micronuclei and cGas/STING activity (57, 58). In agreement, cultured MPB1 tumor cells displayed a trend toward more micronuclei than MP tumor cells, a difference that was exacerbated following cisplatin treatment (Fig. 4*A*). A similar increase was seen in DNA damage as evaluated by γH2AX staining (Fig. 4*B*). These effects correlated with a genotype-specific difference in immune infiltrates of transplanted tumors following cisplatin therapy. Specifically, MPB1 tumors showed a substantial increase in T and NK cells (Fig. 4 *C* and *D*) and a marked reduction of M2-like macrophages compared to MP controls (*SI Appendix*, Fig. S4 *A*–*C*).

To test whether the cGas/STING pathway contributed to the observed genotype-specific effects on drug responses, we generated two independent shRNAs capable of suppressing cGas expression (*SI Appendix*, Fig. S4*D*), transduced these into MPB1 tumor cells, and examined their impact on senescence and tumor phenotypes following cisplatin treatment. While cGAS suppression did not prevent drug-induced proliferative arrest or the appearance of senescence markers (*SI Appendix*, Fig. S4 *E*–*G*), it substantially reduced *Ccl5,* *Cxcl10,* and* Il6* expression (Fig. 4*E* and *SI Appendix*, Fig. S4*H*). In vivo, cGas/STING suppression blunted the therapy-induced accumulation of T and NK cells in transplanted MPB1 tumors (Fig. 4 *F* and *G*), while having no effect on myeloid cell infiltration (*SI Appendix*, Fig. S4 *I*–*K*). These data are consistent with a model whereby preferential induction of cisplatin-induced senescence in *Brca1*-deficient tumor cells contributes to cGas/STING activation and the establishment of a proinflammatory TME.

### *Brca1* Loss Sensitizes Tumors to Chemo and ICB Combination Therapy.

Tumors displaying an inflamed microenvironment often up-regulate molecules that blunt antitumor immunity, a phenomenon that can also occur following cisplatin treatment (59, 60). Accordingly, cisplatin treatment induced cell surface expression of the immune checkpoint molecule PD-L1 on immune and tumor cells in MBP1 (but not MP) tumors (*SI Appendix*, Fig. S5 *A* and *B*). In patients and in a range of preclinical models, such a scenario often predicts increased tumor sensitivity to ICB (61). Accordingly, treatment outcomes were examined in MP or MPB1 EPO-GEMMs or mice harboring syngeneic subcutaneous or i.p. tumors generated by transplantation of EPO-GEMM–derived tumor cells. Of note, the i.p. context mimics the clinically relevant context of disseminated disease after surgical resection of the primary tumor.

**Fig. 5.**
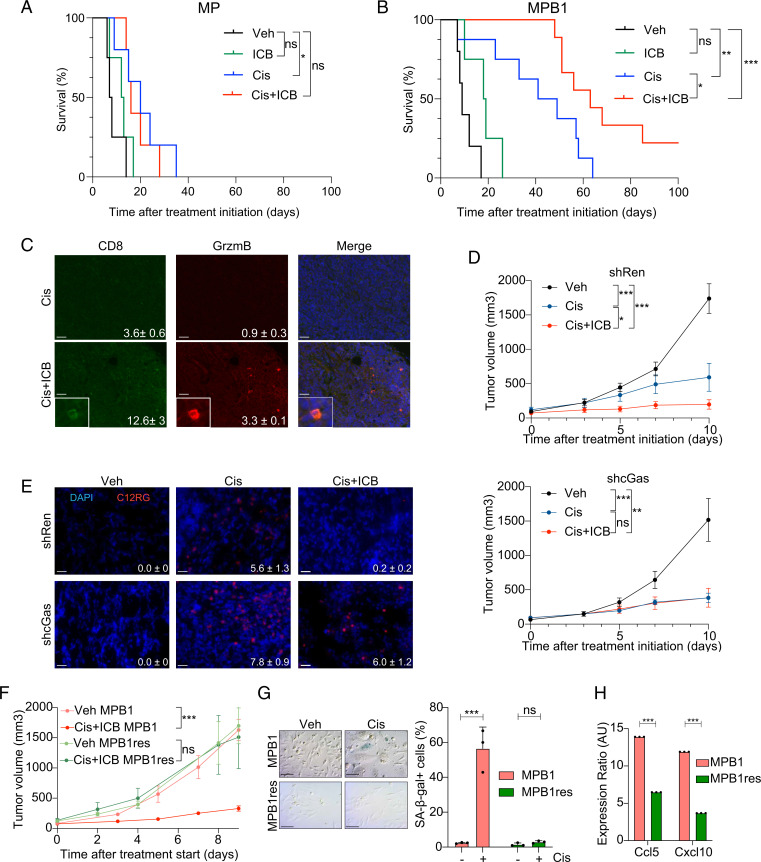
*Brca1* loss sensitizes tumors to chemo and ICB combination therapy. (*A* and *B*) Kaplan–Meier survival curve of MP (*A*) or MPB1 (*B*) EPO-GEMM mice treated with the indicated drugs. Treatment was initiated after tumors were detected by abdominal palpation (*n* = 4 to 9 mice per group). (*C*) Representative IF staining and quantification (mean number of cells per field ± SEM, *P* ≤ 0.05) of subcutaneously transplanted MPB1 ovarian tumors treated with indicated treatments (*n* = 5 fields for two independent tumors). (Scale bar, 20 µm.) (*D*) Tumor growth over time of transplanted MPB1 cell lines containing control Renilla (shRen, *Top*) or cGas shRNAs (shcGas, *Bottom*) with vehicle, cisplatin, or cisplatin + ICB (*n* = 5 to 6 mice per group). (*E*) Staining and quantification of C12RG, a fluorogenic substrate for SA-β-gal activity, in tumors treated as in *D* (*n* = 3). (Scale bar, 20 µm.) (*F*) Tumor growth of transplanted MPB1 or resistant MPB1 (MPB1_res) cell lines treated with vehicle or cisplatin + ICB (*n* = 5 to 6 mice per group). (*G*) SA-β-gal staining (*Left*) and quantification (*Right*) of MPB1 or MPB1_res cell lines treated with vehicle or cisplatin for 6 d (*n* = 3). (Scale bar, 50 µm.) (*H*) RT-qPCR analysis of *Ccl5* and *Cxcl10* in MPB1 or MPB1_res cell lines (*n* = 3 technical replicates). Expression ratio of cisplatin treated relative to vehicle treated is shown. *P ≤ 0.05, **P ≤ 0.01, ***P ≤ 0.001, ns: not significant; mean ± SEM; analyses performed using log-rank test (*A* and *B*), unpaired *t* test (*C* and *E*–*H*), and one-way ANOVA (*D*).

In line with recent findings showing HGSOC patients receive little if any clinical benefit from ICB monotherapy (12), neither MP nor MPB1 tumors showed appreciable responses to anti–PD-1 therapy. In contrast, MPB1 tumors responded more effectively to cisplatin and anti–PD-1 combination therapy compared to MP tumors, in all three tumor settings (Fig. 5 *A* and *B* and *SI Appendix*, Fig. S5 *C*–*F*). This increased responsiveness was associated with an increase in the number of tumor-infiltrating CD8^+^ T cells that expressed the activation marker Granzyme B (Fig. 5*C*). Interestingly, cGas suppression in *Brca1*-deficient tumors curtailed the responsiveness to combination therapy, but not single chemotherapy treatment, resulting in reduced clearance of senescent cells (Fig. 5 *D* and *E*). These data demonstrate that chemo- and immunotherapy uniquely synergize in *Brca1*-deficient EPO-GEMM tumors, whereas cGas/STING activation plays an important role in immune cell recruitment and clearance of senescent cells but is not necessarily required for primary chemotherapy response.

While many ovarian cancer patients initially respond to treatment, most patients eventually develop resistance. To study the process of disease relapse in previously responding tumors, we generated a cell line from a *Brca1*-mutant EPO-GEMM tumor that progressed following treatment with cisplatin and anti–PD-1 antibody and tested responsiveness to cisplatin therapy in vitro and to cisplatin and anti–PD-1 combination therapy in vivo following subcutaneous injection into syngeneic recipients. Interestingly, tumors formed by these cells did not respond to the combination of cisplatin and ICB in vivo (Fig. 5*F*), an effect that correlated with a reduced propensity to undergo senescence (Fig. 5*G*) and induce SASP (Fig. 5*H*). While loss of 53BP1 can restore error-free DNA repair in *Brca1*-mutant cells (62), 53BP1 foci were still induced upon cisplatin treatment (*SI Appendix*, Fig. S5*G*) implying that this mechanism is not responsible for therapy resistance in our system. These data underscore the role of therapy-induced senescence as a mediator of response and resistance to platinum-based chemotherapy in HR-deficient ovarian cancer and its potential to sensitize these tumors to ICB.

## Discussion

HGSOC is a genetically unique tumor type that almost uniformly develops resistance to conventional, targeted, and immune therapies. In this study, we produced a flexible nongermline-based mouse model that recapitulates the genetic, histological, and molecular features of human HGSOC. We illustrate its use for studying genetic interactions during tumorigenesis and exploring molecular mechanisms that dictate treatment response. Our results add to previous work showing that HR defects can produce distinct tumor phenotypes and vulnerabilities (63, 64), and link a chemotherapy-induced senescence program to therapeutic outcome.

We believe that the EPO-GEMM approach described herein provides a platform that will revolutionize preclinical HGSOC research. While traditional germline models offer similar capabilities, they are simply too time consuming and asynchronous to be a workhorse system. By contrast, the EPO-GEMM approach enables the production of autochthonous tumors in immunocompetent mice that naturally disseminate, enabling the relatively synchronous production of cohorts of tumor-bearing mice simply from a set of plasmids and commercially available immunocompetent WT mice. While the use of lineage-specific Cas9 or CRE transgenes allows for the control of the cell of origin, electroporation of the ovary and fallopian tube of WT mice produces epithelial tumors that resemble human HGSOC, similar to what was observed in cell line studies (28). As such, the approach can be applied to any strain of recipient mice, enabling the study of the influence of host factors on tumor trajectories.

Our study is complementary to a recent report that also used tissue electroporation to generate immune-competent ovarian cancer models (65). While the methods used in both studies are conceptually similar, they differ in the choice of oncogenic lesions, latency, and spontaneous metastatic spread in WT hosts. Furthermore, our study incorporates transgene vectors for oncogene expression and confirms that the resulting tumors are of epithelial origin and display molecular similarities to the human disease. By incorporating *MYC* overexpression and *Brca1* disruption into the platform, we substantially accelerate tumor onset and enable modeling of clinically important HR-deficient tumors. Collectively, these approaches provide a powerful orthogonal system to ovarian cancer models produced from tumor-derived cell or organoid lines (15–19, 66–68).

Our results implicate a cellular senescence program as an important component of response and resistance in HGSOC. Previous work suggests that senescence-inducing therapeutics can stimulate a SASP-dependent remodeling of the TME that, in some instances, leads to senescent cell clearance or sensitizes tumor cells to immune recognition following checkpoint blockade (44, 45). By contrast, in other settings, treatment-associated SASP programs can stimulate tumor relapse and dissemination (69). Herein, we show that the ability of cisplatin or taxol to induce senescence in ovarian cancer cells depends on tumor genotype, being substantially more pronounced following treatment of *Brca1*-deficient (compared to *Brca1*-proficient) ovarian tumors. In turn, this leads to an increase in micronuclei and a cGas/STING-mediated SASP that alters immune cell infiltrates and sensitizes the *Brca1*-deficient tumors to ICB.

Consistent with the importance of the senescence program in therapy response, senescence signatures can be detected in posttreatment samples from HGSOC patients. Moreover, tumors derived from a *Brca1*-deficient cancer that progressed on treatment lose their ability to induce senescence and SASP upon cisplatin treatment, leading to resistance to cisplatin in combination with ICB. Interestingly, the increased propensity of *Brca1*-deficient tumors to undergo senescence and/or activate the cGas/STING pathway appears to extend to other agents, as *Brca1*-deficient models of breast and ovarian cancer treated with PARP inhibitors or, as shown here, taxol show similar behaviors (70–74). As such, senescence induction may underlie the improved response of HR-deficient tumors to genome destabilizing therapies in the clinic.

In our system, the SASP program triggered by cisplatin therapy in *Brca1*-deficient ovarian tumors was limited to *Ccl5*, *Cxcl10*, and *Il6* of the factors examined. cGas suppression efficiently suppressed SASP induction and sensitization to ICB following cisplatin treatment yet had no effect on treatment outcomes following cisplatin monotherapy. This implies that SASP is sufficient to sensitize tumor cells to ICB and, in agreement, injection of SASP-activated tumor cells sensitizes an immunologically cold murine ovarian model to ICB (75), whereas CCL5 suppression in another model attenuates T cell inflammation (73). While it remains possible that senescence and cGas/STING-dependent cytokine induction are parallel processes, they imply that potent antitumor responses require both cell-intrinsic senescence induction and TME modulation.

Although HR deficiency can increase tumor immunogenicity (76), *BRCA1/2* mutations have no effect on the response to ICB monotherapy in HGSOC patients (6, 7, 12). Our model recapitulates these findings: *Brca1*-deficient tumors displayed an increase in immune infiltration pretreatment yet are nonresponsive to ICB. By contrast, our results imply that frontline chemotherapy or PARP inhibitors should sensitize HR-deficient tumors to checkpoint blockade, yet clinical trials to date fail to validate such hypersensitivities as universally operative in patients (77–80). Instead, these trials identify tumor positivity for PD-L1 and CD8 expression—features of the *Brca1*-deficient tumors studied herein—as biomarkers of a combinatorial response (81). It seems likely that the disparate outcomes between the human and animal studies reflect the longer course of tumor evolution in patients, which may further degrade components of the senescence machinery. Accordingly, we see that *Brca1*-deficient tumor cells that acquire senescence defects are nonresponsive to the chemotherapy/ICB combination. Future studies incorporating the flexible features of the EPO-GEMM approach will enable the further dissection of mechanisms that dictate ovarian cancer response and resistance and, more broadly, expedite investigation of other clinically relevant aspects of this disease.

## Materials and Methods

Below is an abbreviated summary of the materials and methods used. More details can be found in *SI Appendix*, *Supplementary Methods*.

### Generation of EPO-GEMMs.

The 8- to 12-wk-old WT C57BL/6, or transgenic CK8-CreER;LSL-Cas9-IRES-GFP female mice, were anesthetized with isofluorane, and the surgical site was scrubbed with a povidone-iodine scrub (Betadine) and rinsed with 70% alcohol. The target organ was accessed from the left flank, as this allowed for the more readily stabilization of the organ for electroporation than accessing it from the back. After opening the skin and peritoneum, the left ovary and oviduct were exteriorized. A total of 25 μL of a plasmid mix (details in *SI Appendix*, Table S1) was injected under the ovarian bursa using a 30-gauge syringe, which led to the formation of a round, liquid-containing bubble. Tweezer electrodes were tightly placed around this “injection bubble.” Two poring pulses of electrical current (50 V) given for 30-ms lengths at 450-ms intervals and five transfer pulses (60 V, 50-ms length, 450-ms intervals) were then applied using an in vivo electroporator (NEPAGENE NEPA21 type II electroporator) (22). After successful electroporation, the peritoneal cavity was rinsed with 500 µL of prewarmed saline. Then, the peritoneal cavity was sutured, and skin staples were used to close the skin. Until they awoke, mice were kept at 37 °C, and postsurgery pain management was done with buprenorphine injections for 3 d. Tumor formation was assessed by abdominal palpation and ultrasound imaging. Tumors were isolated at a humane endpoint.

### Characterization of EPO-GEMM Tumors.

Histopathological features of EPO-GEMM primary tumors and metastases were assessed by a trained veterinary pathologist (J.E.W.) and their relationship to human HGSOC was determined by immunohistochemistry for relevant markers and through bulk RNA sequencing of tumor tissue. Tumors were shown to harbor intended lesions using Sanger sequencing of the CRISPR-Cas9–induced scar and immunoblotting for MYC. Tumor clonality was analyzed using next-generation DNA sequencing of the *Trp53* amplicon and sparse whole-genome sequencing was used to characterize CNAs (32, 82). Flow cytometry was performed to evaluate tumor immune infiltration.

### EPO-GEMM Cell Line Generation.

For cell line generation, a tumor piece was minced with a razorblade into small pieces, placed in 5 mL of prewarmed collagenase V buffer (1 µg/mL, Sigma-Aldrich), and incubated at 37 °C for 30 min. Dissociated tissue was washed once with phosphate buffered saline, filtered through a 70-µm cell strainer and centrifuged at 1,500 rpm for 5 min. Cells were plated on 10-cm culture dishes coated with 100 µg/mL collagen (PureCol 5005; Advanced Biomatrix). Primary cultures were passaged at least three times to remove fibroblast contamination. All ovarian cancer cell lines were maintained in a humidified incubator at 37 °C with 5% CO_2_ and grown in Dulbecco's modified Eagle's medium supplemented with 10% fetal bovine serum and 100 IU/mL penicillin/streptomycin. All cell lines used in this study tested negative for *Mycoplasma*. Cell lines were validated to carry the correct genotype and to have tumor-initiating capabilities following subcutaneous and i.p. injection. Multiple tumor-derived cell lines were confirmed to produce consistent treatment response patterns in vitro.

### Characterization of Cellular Senescence and cGas/STING Response.

Assays to evaluate cellular senescence involved SA-β-gal staining (42) and replating assays after drug withdrawal. SASP profiles were assessed using murine cytokine arrays (Eve Technologies) and RT-qPCR. Micronuclei were visualized and quantified by nuclear DAPI staining. The DNA-damage response was determined using IF for 53BP1, γH2AX, and Rad51 (18). The role of the cGas/STING pathway was assessed by transducing cells with two independent cGas shRNAs validated for knockdown and compared to a well-established control shRNA.

### Human Cell Line and Tumor Analyses.

UWB1.289, UWB.289 + BRCA1, MDA-MB-231, and MDA-MB-436 cell lines were purchased from American Type Culture Collection and cultured according to instructions. CBioPortal.org was used to plot the frequency of mutations, amplifications, and/or deletions in genes of interest in HGSOC patients from various datasets. To evaluate senescence signatures in human tumor samples, senescence signatures were derived from KEGG and previously published works (44, 83). TPMs (transcripts per million) normalized expression data were used to calculate geometric mean score as the senescence signature scores.

### Statistics.

Statistical analyses were performed using Prism 6 software (GraphPad Software) as described in the figure legends. Statistically significant differences (*P* < 0.05) are indicated with asterisks, accompanied by *P* values in the legends. Statistical significance was determined by Student’s *t* test, one-way ANOVA, log-rank test, Pearson’s correlation, or Wilcoxon signed-rank test. Survival was measured using the Kaplan–Meier method. Error bars indicate SEM. Unless otherwise stated, the indicated sample size (*n*) represents biological replicates. All samples that met proper experimental conditions were included in the analysis.

## Supplementary Material

Supplementary File

## Data Availability

RNA-sequencing data have been deposited in Gene Expression Omnibus (GSE181651).
