# Supplementary material for: Senescence induction dictates response to chemo- and immunotherapy in preclinical models of ovarian cancer
Source: Proc Natl Acad Sci U S A. 2022 Jan 26;119(5):e2117754119. doi: 10.1073/pnas.2117754119 (PMC8812522; doi:10.1073/pnas.2117754119)
Supplement: Supplementary File [file pnas.2117754119.sapp.pdf]

Supplementary Figures

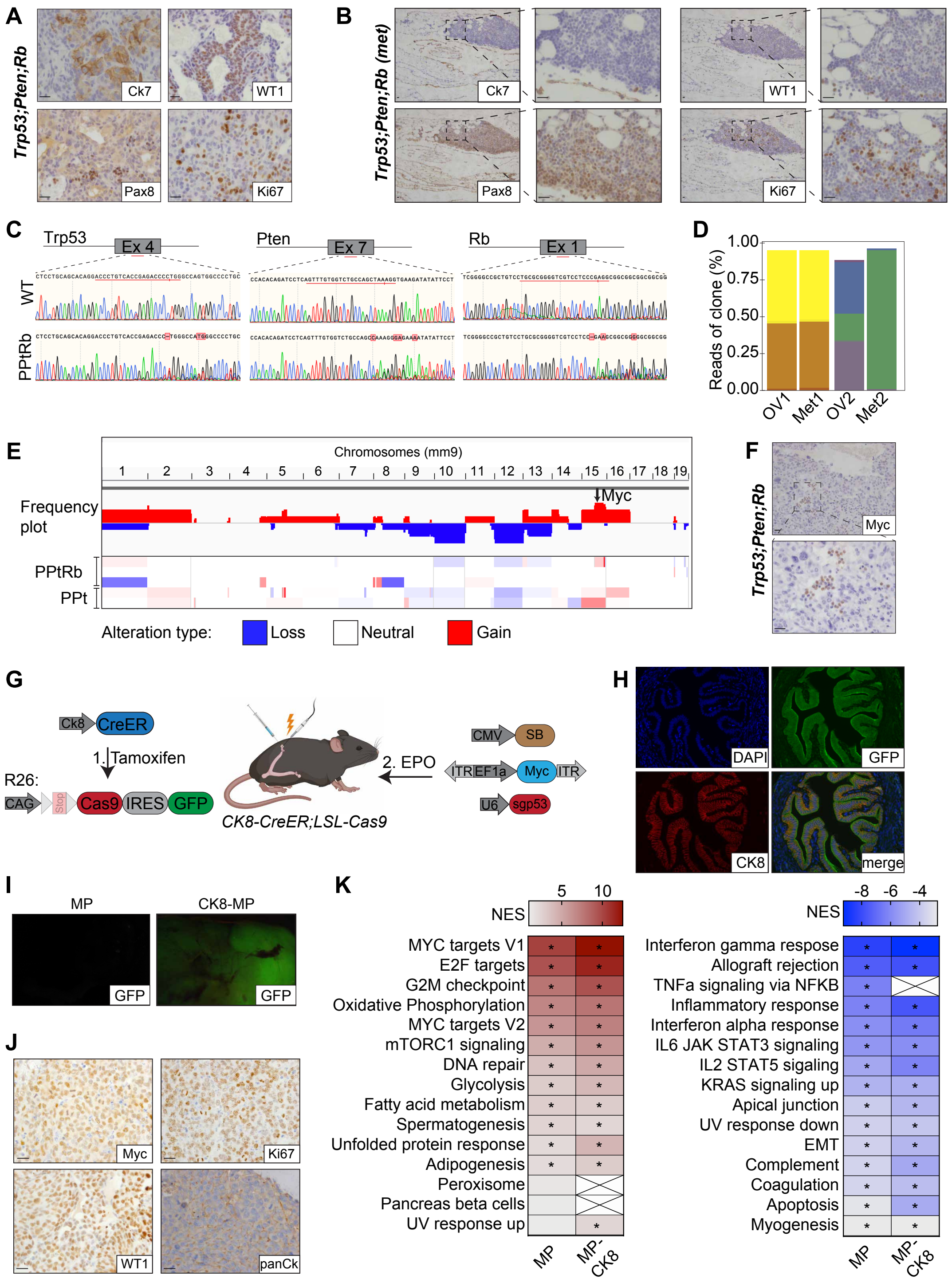

Figure S1: EPO-GEMM approach generates high-grade serous ovarian cancer that is of epithelial origin. (A-B) Representative immunohistochemical staining of a *Trp53;Pten;Rb* EPO-GEMM ovarian tumor **(A)** and a corresponding micrometastasis in the omentum **(B)** for the HGSOC markers CK7, WT1, Pax8 and Ki67. Scale bar 20 µm. **(C)** Sanger sequencing confirming editing of the respective gene loci targeted by the indicated CRISPR-Cas9-sgRNAs in a *Trp53;Pten;Rb* EPO-GEMM ovarian tumor compared to an unmodified wild-type (WT) tissue. **(D)** Clonality analysis of two representative paired primary tumors (OV) and omentum metastasis (Met). Percentage of reads of the dominant clones was derived by deep sequencing of the *Trp53* amplicon. **(E)** Frequency plot of CNA analysis of *Trp53;Pten;Rb* (PPtRb, n=3) and *Trp53;Pten* (PPt, n=2) EPO-GEMM ovarian tumors. **(F)** Representative immunohistochemical staining of a *Trp53;Pten;Rb* EPO-GEMM ovarian tumor for MYC. Scale bar 20 µm. **(G)** Schematic of the EPO-GEMM approach *in CK8-CreER;LSL-Cas9-IRES-GFP* mice. Tamoxifen is given to excise the Stop-cassette to drive Cas9 expression in Ck8-positive cells. A *MYC* transposon vector, a transposon vector harboring a sgRNA targeting *Trp53* (sg*p53*), and a Sleeping Beauty transposase (SB) are delivered into the ovary of *CK8-CreER-Cas9* mice by direct *in vivo* electroporation. **(H)** Representative immunofluorescence staining of the oviduct of a *CK8-CreER;LSL-Cas9-IRES-GFP* mouse one week after Tamoxifen treatment. **(I)** Macroscopic GFP expression in MP tumors generated by *in vivo* tissue electroporation of a WT mouse (left) or a *CK8-CreER;LSL-Cas9-IRES-GFP* mouse (right). **(J)** Representative immunohistochemical staining of MP EPO-GEMM ovarian tumors generated in *CK8-CreER;LSL-Cas9-IRES-GFP* mice. Scale bar 20 µm. **(K)** Comparison of top enriched (left, red) and depleted (right, blue) Hallmark genesets derived from RNA-seq data in MP and CK8-MP EPO-GEMM tumors compared to normal tissue. Star indicates p-value < 0.05.

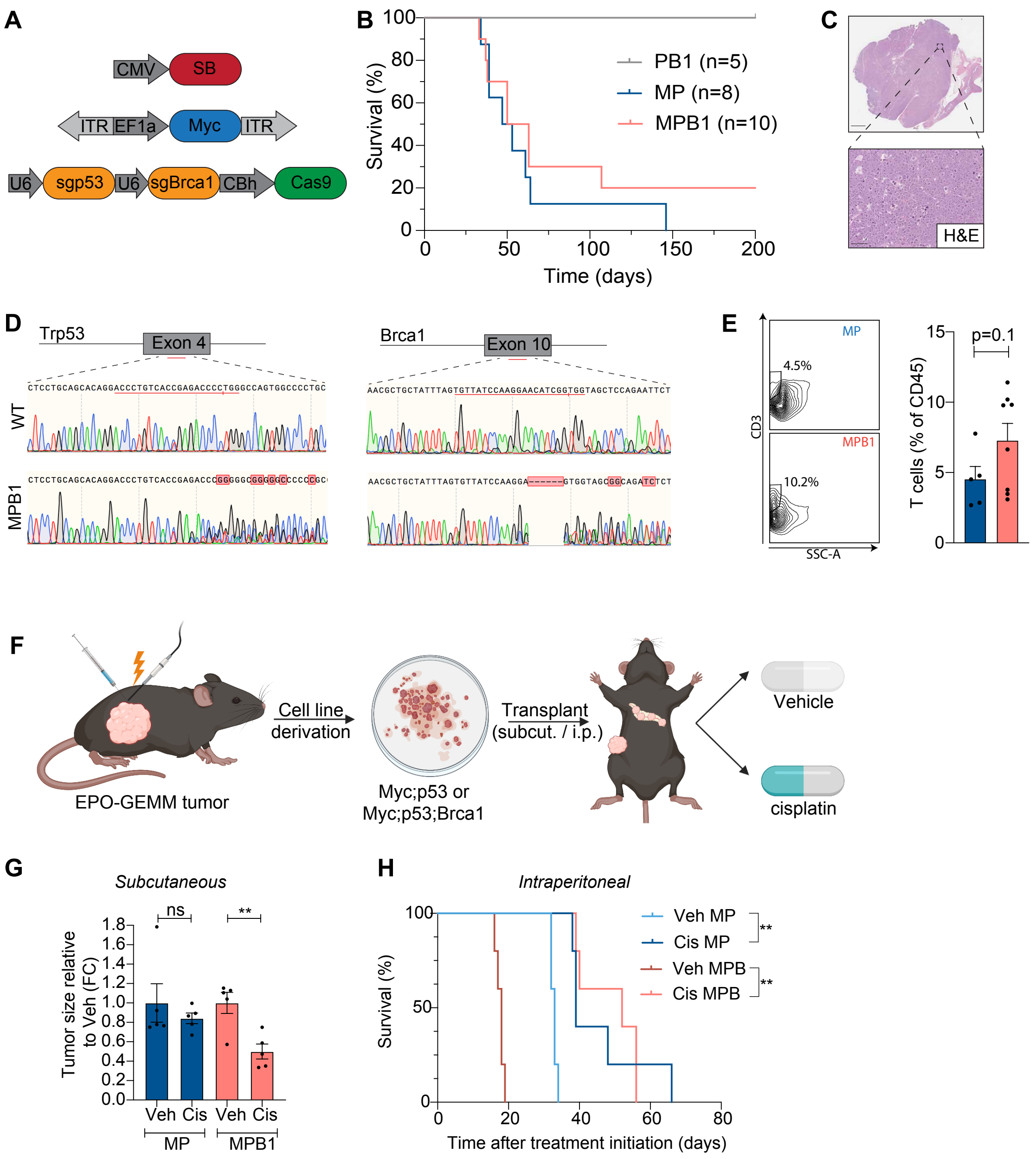
A
CMV
SB
ITR
EF1a
Myc
ITR
U6
sgp53
U6
sgBrca1
CBh
Cas9
B
Survival (%)
Time (days)
PB1 (n=5)
MP (n=8)
MPB1 (n=10)
C
H&E
D
Trp53
Exon 4
Brca1
Exon 10
WT
MPB1
E
MP
4.5%
MPB1
10.2%
CD3
SSC-A
p=0.1
T cells (% of CD45)
F
EPO-GEMM tumor
Cell line derivation
Myc;p53 or Myc;p53;Brca1
Transplant (subcut. / i.p.)
Vehicle
cisplatin
G
Subcutaneous
Tumor size relative to Veh (FC)
ns
**
Veh
Cis
Veh
Cis
MP
MPB1
H
Intraperitoneal
Survival (%)
Veh MP
Cis MP
Veh MPB
Cis MPB
**
**
Time after treatment initiation (days)

Figure S2: HR deficiency drives tumors with increased T-cell infiltration and improved therapy response. (A) Plasmid combination used to induce HR-deficient EPO-GEMM tumors. **(B)** Kaplan-Meier survival curve of C57BL/6 mice electroporated with the indicated combinations of plasmids. **(C)** Representative H&E staining of a MPB1 EPO-GEMM tumor. Scale bar 5000 µm (top), 100 µm (bottom). **(D)** Sanger sequencing confirming editing of the *Trp*53 and *Brca1* gene loci targeted by the indicated CRISPR-Cas9 sgRNAs in a MPB1 EPO-GEMM ovarian tumor. **(E)** Analysis of the T cell infiltrate of representative EPO-GEMM tumors of the indicated genotypes by flow cytometry (n=5-8 mice per group). **(F)** Schematic of transplantation approach of EPO-GEMM derived cell lines. **(G)** Relative tumor size of subcutaneously transplanted MP or MPB1 tumor cell lines treated with vehicle or cisplatin (n=5 mice per group). **(H)** Kaplan-Meier survival curve of mice after i.p. transplantation of MP or MPB1 tumor cell lines and treatment with vehicle or cisplatin (n=5 mice per group). Mice were randomized according to luciferase signal before treatment initiation.

*p ≤ 0.05, **p ≤ 0.01, ***p ≤ 0.001, ns: not significant; Mean ± SEM; Analyses performed using unpaired t test (E, G) and log-rank test (H).

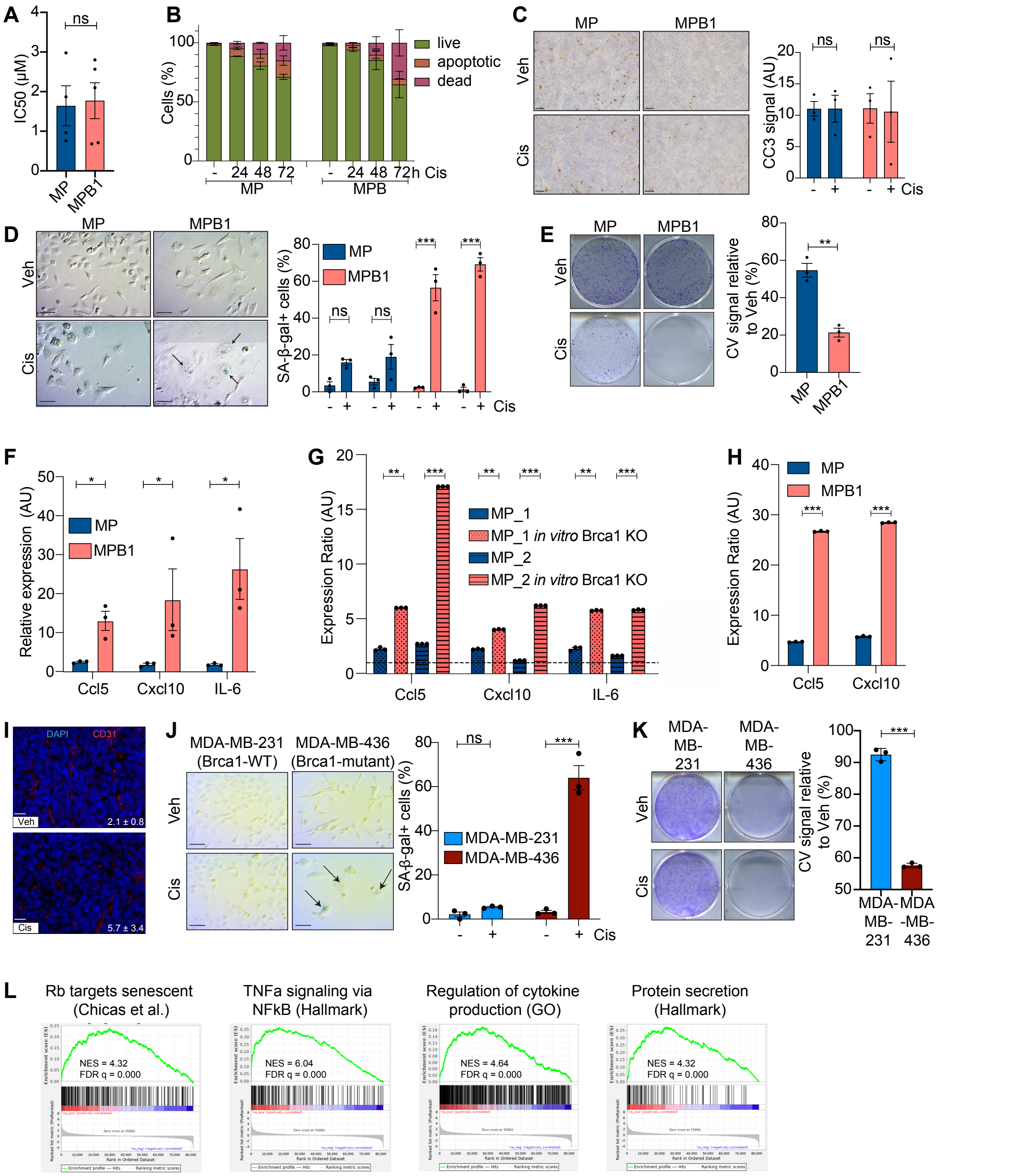

Figure S3: Chemotherapy treatment induces senescence and SASP in HR-deficient HGSOC. **(A)** IC50 values of MP or MPB1 cell lines treated with cisplatin. Cell viability was calculated relative to vehicle-treated control cells, measured with CellTiter-Glo assay 72h after treatment (n = 4-5). **(B)** Quantification of live (Annexin-V$^-$, PI$^-$), apoptotic (Annexin-V$^+$, PI$^-$) or dead (Annexin-V$^+$, PI$^+$) cells in MP or MPB1 cell lines after 24, 48 or 72 h of cisplatin treatment (n = 3). **(C)** Representative

immunohistochemical staining and quantification of cleaved caspase-3 (CC3) signal in subcutaneously transplanted MP or MPB1 ovarian tumors after two cycles of cisplatin treatment (n = 3). Scale bar 40 µm. **(D)** SA-ß-gal staining of cell lines treated with vehicle or 1 µM cisplatin for 6 days (n = 2 independent cell lines per genotype with n = 3 technical replicates). Scale bar 50 µm. **(E)** Clonogenic crystal violet (CV) assay of MP or MPB1 cells replated in the absence of drugs after 6-day pretreatment as in (D) (n = 3 independent cell lines per genotype). **(F)** RT-qPCR analysis of *Ccl5*, *Cxcl10* and *Il6* in MP or MPB1 cell lines. Expression ratio of cisplatin-treated relative to untreated is shown. Each point represents a cell line derived from a different mouse tumor (n = 3). **(G)** RT-qPCR analysis of *Ccl5*, *Cxcl10* and *Il6* in MP cell lines transfected with control or *Brca1*-targeting sgRNA. Expression ratio of cisplatin-treated relative to untreated is shown. The different patterns represent a cell line derived from a different MP mouse tumor, in which *Brca1* was knocked-out after cell line establishment (n = 3 technical replicates per independent line). **(H)** RT-qPCR analysis of *Ccl5* and *Cxcl10* in MP or MPB1 cell lines treated with 50 nM Taxol. Expression ratio of treated relative to untreated is shown (n = 3). **(I)** IF staining and quantification of $CD31^+$ blood vessels of transplanted tumors after two cycles of cisplatin treatment (n = 3). Scale bar 20 µm. **(J)** SA-ß-gal staining (left) and quantification (right) of either *BRCA1* wild-type (WT) (MDA-MB-231) or -mutant (MDA-MB-436) human breast cancer cell lines after treatment with vehicle or cisplatin for 6 days (n = 3). Scale bar 50 µm. **(K)** Clonogenic crystal violet (CV) assay of human BRCA-WT or -mutant breast cancer cells replated in the absence of drugs after 6-day pretreatment as in (J) (n = 3). **(L)** Gene set enrichment analysis (GSEA) comparing expression of senescence and SASP signatures in patients after and before chemotherapy treatment in a human ovarian cancer dataset (*1*).

*p ≤ 0.05, **p ≤ 0.01, ***p ≤ 0.001, ns: not significant; Mean ± SEM; Analyses performed using unpaired t-test (A, C, E-K), one-way ANOVA (D) and Wilcoxon signed-rank test (L).

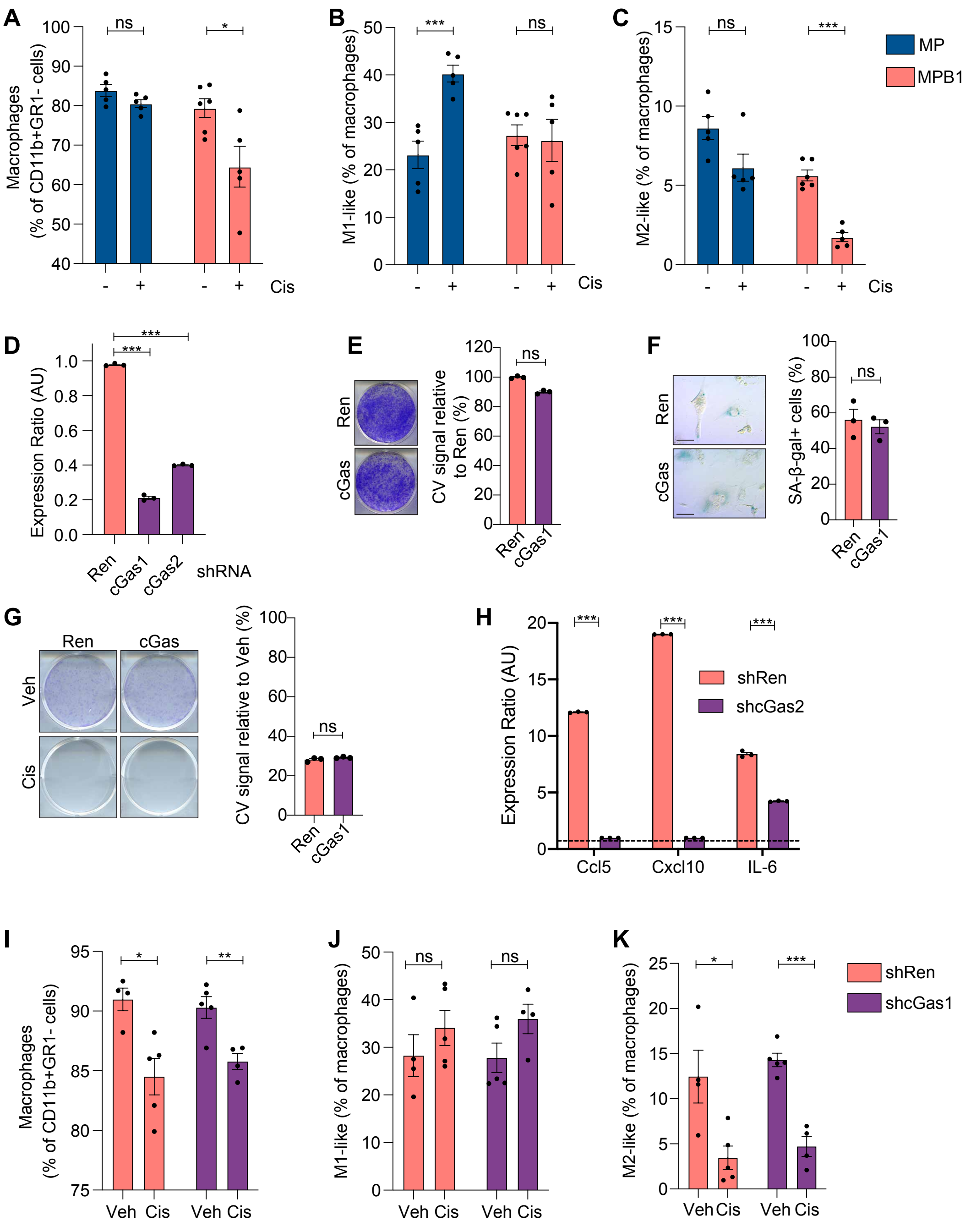
A
Macrophages (% of CD11b+GR1- cells)
100
90
80
70
60
50
40
ns
*
- + - + Cis
B
M1-like (% of macrophages)
50
40
30
20
10
0
***
ns
- + - + Cis
C
M2-like (% of macrophages)
15
10
5
0
ns
***
- + - + Cis
MP
MPB1
D
Expression Ratio (AU)
1.0
0.8
0.6
0.4
0.2
0.0
***
***
Ren
cGas1
cGas2
shRNA
E
Ren
cGas
CV signal relative to Ren (%)
120
100
80
60
40
20
0
ns
Ren
cGas1
F
Ren
cGas
SA-β-gal+ cells (%)
100
80
60
40
20
0
ns
Ren
cGas1
G
Ren
cGas
Veh
Cis
CV signal relative to Veh (%)
100
80
60
40
20
0
ns
Ren
cGas1
H
Expression Ratio (AU)
20
15
10
5
0
***
***
***
shRen
shcGas2
Ccl5
Cxcl10
IL-6
I
Macrophages (% of CD11b+GR1- cells)
95
90
85
80
75
*
**
Veh Cis Veh Cis
J
M1-like (% of macrophages)
50
40
30
20
10
0
ns
ns
Veh Cis Veh Cis
K
M2-like (% of macrophages)
25
20
15
10
5
0
*
***
Veh Cis Veh Cis
shRen
shcGas1

Figure S4: cGas knockdown does not alter the myeloid immune compartment or cell-intrinsic senescence response. (A-C) Infiltration of macrophages (**A**) and percentages of M1-like (CD206-, CD80+) (**B**) and M2-like (CD206+, CD80-) (**C**) macrophages in subcutaneously transplanted MP or MPB1 ovarian tumors after treatment with 2 cycles of cisplatin (n = 5-6 mice per group). **(D)** RT-qPCR analysis of cGas in MPB1 cell lines containing control Renilla (shRen) or cGas shRNAs (shcGas) shRNAs targeting (n = 3). **(E)** Clonogenic assay of MPB1 cell lines containing control Renilla (shRen) or cGas (shcGas) shRNAs (n = 3). **(F)** SA-ß-gal staining of MPB1 cell lines containing control Renilla (shRen) or cGas (shcGas) shRNAs after treatment with vehicle or cisplatin for 6 days (n = 3). Scale bar 20 µm. **(G)** Clonogenic crystal violet (CV) assay of MP or MPB1 cells replated in the absence of drugs after 6-day pretreatment as in (F) (n = 3). (**H**) RT-qPCR analysis of *Ccl5*, *Cxcl10* and *Il6* in MPB1 cell lines containing control Renilla (shRen) or cGas (shcGas) shRNAs. Expression ratio of cisplatin-treated relative to untreated is shown (n = 3). (**I-K**) Infiltration of macrophages (**I**) and percentages of M1 (**J**) and M2 (**K**) macrophages in transplanted shRen or shcGas MPB1 ovarian tumors after treatment with 2 cycles of cisplatin (n = 4-5 mice per group).

*p ≤ 0.05, **p ≤ 0.01, ***p ≤ 0.001, ns: not significant; Mean ± SEM; Analyses performed using unpaired t-test (A-C, E-K) or one-way ANOVA (D).

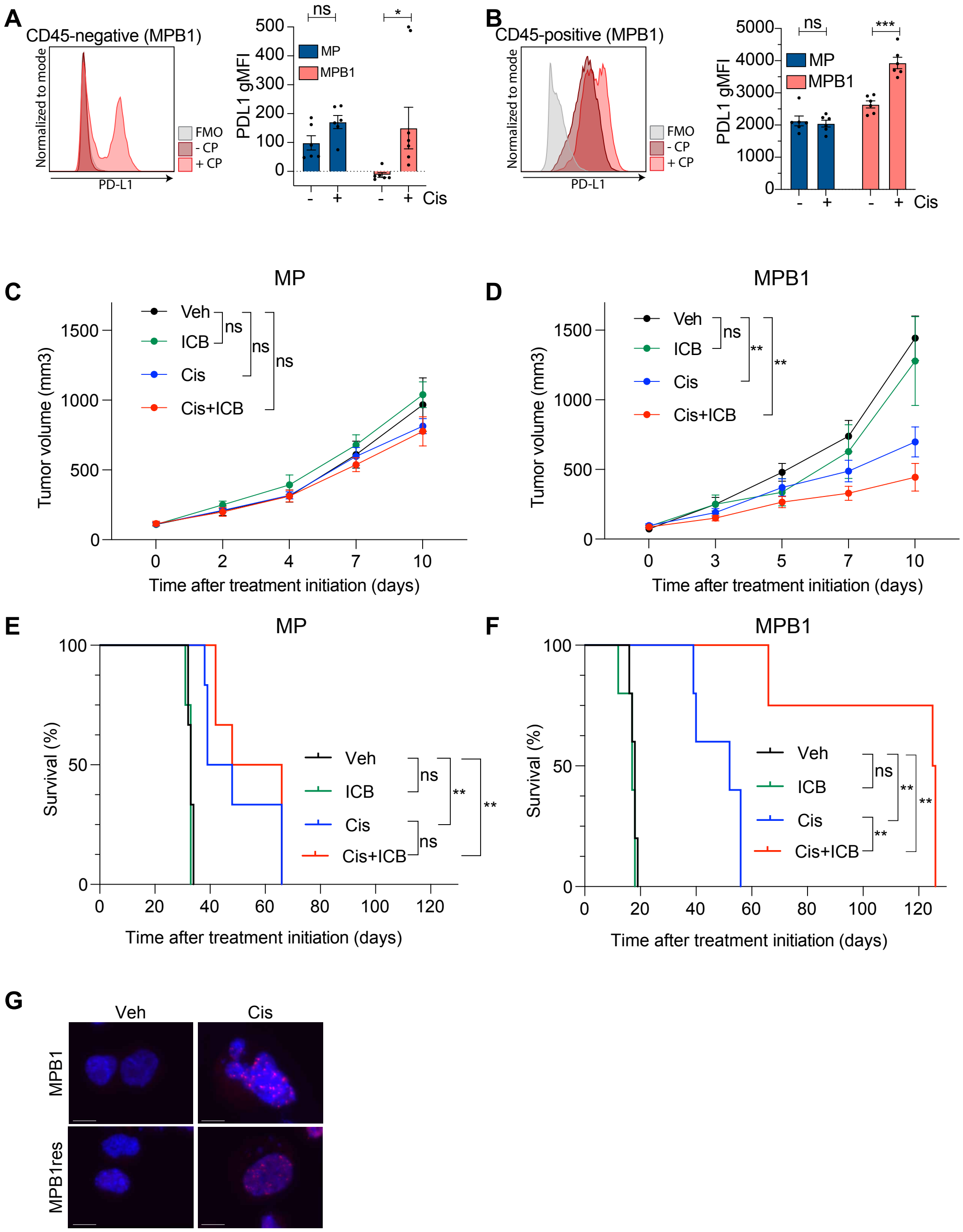
A
CD45-negative (MPB1)
Normalized to mode
PD-L1
FMO
- CP
+ CP
PDL1 gMFI
MP
MPB1
ns
*
Cis
B
CD45-positive (MPB1)
C
MP
Veh
ICB
Cis
Cis+ICB
Tumor volume (mm3)
Time after treatment initiation (days)
D
MPB1
E
Survival (%)
F
G
Veh
Cis
MPB1
MPB1res

Figure S5: Treatment response of HR-proficient and -deficient HGSOC after treatment with chemotherapy and ICB. (A+B) Representative MFI plot (left) and quantification (right) of PD-L1 expression on tumor cells (CD45-negative) **(A)** or immune cells (CD45-positive) **(B)** from subcutaneously transplanted MPB1 ovarian tumors treated with vehicle or cisplatin (n = 5-6 mice per group). **(C+D)** Tumor growth of subcutaneously transplanted MP **(C)** or MPB1 **(D)** tumors treated with vehicle, ICB, cisplatin or cisplatin + ICB (n = 5 mice per group). **(E+F)** Kaplan-Meier survival curve of MP **(E)** or MPB1 **(F)** tumors generated by i.p. injection (n = 4-6 mice per group). Mice were randomized according to luciferase signal before treatment initiation. **(G)** Immunofluorescence staining of 53BP1 foci in MP and MPB1 cell lines treated with vehicle or cisplatin for 72h. Scale bar 10 µm.

*p ≤ 0.05, **p ≤ 0.01, ***p ≤ 0.001, ns: not significant; Mean ± SEM; Analyses performed using unpaired t-test (A-B), one-way ANOVA (C-D) and log-rank test (E-F).

Supplementary Methods

Animal studies

Mice were maintained under specific pathogen-free conditions, and food and water were provided ad libitum. Mice were purchased from Jackson laboratory. CK8-CreER (*2*) male mice were crossed with LSL-Cas9-IRES-GFP female mice to produce CK8-CreER;LSL-Cas9-IRES-GFP female mice for generation of EPO-GEMMs. All mouse experiments were approved by the Memorial Sloan-Kettering Cancer Center (MSKCC) Internal Animal Care and Use Committee.

Ultrasound and bioluminescence imaging

High-contrast ultrasound imaging was performed on a Vevo 2100 System with a MS250 13- to 24-MHz scanhead (VisualSonics) to stage and quantify ovarian EPO-GEMM tumor burden. Tumor volume was analyzed using Vevo LAB software.

For visualizing ovarian tumor cells with luciferase, luciferase-blasticidin (Luc-Blast) constructs were cloned into MSCV-based vectors and retroviruses were packaged by co-transfection of Gag-Pol expressing 293 T cells with expression constructs and envelope vectors (VSV-G) using the Lipofectamine method (Thermo Fisher). Following transduction, cells were selected with Blasticidin S (10 μg/ml; Life Technologies) for 5 days. Bioluminescence imaging was used to track luciferase expression in tumor cells expressing the Luc-Blast reporter. Mice were injected i.p. with luciferin (5 mg/mouse; Gold Technologies) and then imaged on a Xenogen IVIS Spectrum imager (PerkinElmer) 10 minutes later for 30 s. Quantification of luciferase signaling was analyzed using Living Image software (Caliper Life Sciences).

Preclinical drug studies

For preclinical treatment studies, EPO-GEMM mice were monitored for tumor development by palpation or ultrasound and randomized into treatment groups. For subcutaneous studies, EPO-GEMM derived cell lines were resuspended in Matrigel (BD Biosciences) and injected in the subcutaneous space. Following inoculation, mice were monitored three times a week. Caliper measurements began when tumors became visible. Tumor volume was calculated using the

following formula: tumor volume = (D x d^2)/2, in which D and d refer to the long and short tumor diameter, respectively. When tumors reached a size of 100-150 mm^3, mice were randomized based on starting tumor volume and enrolled into treatment groups. Tumor size and mouse weights were recorded three times weekly. Experimental endpoints were achieved when tumors reached 2000 mm^3 or became ulcerated. For i.p. studies, 1 to 2.5 million cells of EPO-GEMM derived cell lines carrying a luciferase reporter were resuspended in PBS and injected into the i.p. space in a volume of 200 µl. Tumor volume was monitored using IVIS imaging and mice were randomized based on starting tumor signal.

Mice were treated with vehicle or cisplatin (3 mg/kg body weight) by i.p. injection once a week. Anti-PD-1 antibody (200mg/mouse; RMP1-14, BioXCell) was given 3 times per week i.p. alone or in combination with cisplatin. No obvious toxicities were observed in vehicle- or drug-treated animals as assessed by changes in body weight. Upon sacrifice, ovarian tumor tissue was allocated to either 10% formalin fixation, flow cytometry analysis on fresh tissue or snap frozen for DNA/RNA analysis.

DNA constructs for electroporation

The Sleeping Beauty transposase (SB13) and the pT3 transposon vector were a generous gift of Dr. Xin Chen, UCSF San Francisco. The pX330 vector was a gift from Feng Zhang (Addgene plasmid # 42230). Table S2 provides the sgRNA sequences used in this study.

Clonality analysis of EPO-GEMM tumors

Genomic DNA was isolated from EPO-GEMM tumors using QIAGEN DNeasy Blood and Tissue kit following manufacturer's instructions. The p53 locus was amplified using a 50 µl reaction following standard Q5 High Fidelity Master Mix (NEB) protocol (forward primer: CAGAAGATATCCTGGTAAGG, reverse primer: CTACAGGCTGAAGAGGAACC). Amplicons were confirmed on a 2% agarose gel and PCR purified using QIAGEN QIAquick PCR purification kit. DNA concentration were measured using Nanodrop and samples were normalized to 20 ng/µl

and sequenced using EZ-amplicon sequencing (MiSeq, 2 x 250 bp by GENEWIZ, Inc, South Plainfield, NJ, USA).

CNA inference

1 µg of bulk genomic DNA (gDNA) was extracted from ovarian tumors and tissue using the DNeasy Blood & Tissue Kit (Qiagen) and sonicated using the Covaris instrument. Sonicated DNA was subsequently end-repaired/A-tailed, followed by ligation of TruSeq dual indexed adaptors. Indexed libraries were enriched via PCR and sequenced in multiplex fashion using the Illumina HiSeq2500 instrument to achieve roughly 1 million uniquely mappable reads per sample – a read count sufficient to allow copy number inference to a resolution of approximately 400kb. For data analysis, uniquely mapped reads were counted in genomic bins corrected for mappability. Read counts where subsequently corrected for GC content, normalized, and segmented using Circular Binary Segmentation (CBS). Segmented copy number calls are illustrated as relative gains and losses to the median copy number of the entire genome.

Tumor RNA-sequencing (RNA-seq)

For RNA-seq analysis of the transcriptional profiles of EPO-GEMM ovarian tumors, as well as normal ovaries of WT C57BL/6 mice, total RNA was extracted from bulk tissue using the RNeasy Mini Kit (Qiagen). Purified polyA mRNA was subsequently fragmented, and first and second strand cDNA synthesis performed using standard Illumina mRNA TruSeq library preparation protocols. Double stranded cDNA was subsequently processed for TruSeq dual-index Illumina library generation. For sequencing, pooled multiplexed libraries were run on a HiSeq 2500 machine on RAPID mode. Approximately 10 million 76bp single-end reads were retrieved per replicate condition. Resulting RNA-seq data was analyzed by removing adaptor sequences using Trimmomatic (*3*), aligning sequencing data to GRCm38.91(mm10) with STAR (*4*), and genome wide transcript counting using featureCounts (*5*) to generate a TPM matrix of transcript counts. Genes were identified as differentially expressed using R package DESeq2 with a cutoff of absolute log2(fold change) ≥ 1 and adjusted p-value < 0.05 between experimental conditions (*6*).

Clustering and Gene Set Enrichment Analysis (GSEA)

Principal component analysis was performed using the DESeq2 package in R. Gene expressions of RNA-seq data were clustered using hierarchical clustering based on one minus pearson correlation test. For pathway enrichment analysis, the weighted GSEA Preranked mode was used on a set of curated signatures in the molecular signatures database (http://www.broadinstitute.org/gsea/msigdb/index.jsp, MSigDB v7.0). From 22,596 signatures, signatures with 15-500 genes were considered for further analyses. From the results, enriched signatures with an adjusted p-value less than 0.05 were considered as statistically significant. Hallmark and Kegg pathways were used to run GSEA on our murine EPO-GEMM models and human patient data (*7*), and $-\log_{10}$(FDR) values were plotted in the XY plot.

Immunohistochemistry and immunofluorescence

Tissues were fixed overnight in 10% formalin, embedded in paraffin, and cut into 5 μm sections. Haematoxylin and eosin (H&E), immunohistochemical and immunofluorescence stainings were performed using standard protocols. Sections were de-paraffinized, rehydrated, and boiled in a microwave for 15 minutes in 10 mM citrate buffer (pH 6.0) for antigen retrieval. Antibodies were incubated overnight at 4ºC. Primary antibodies are listed in Table S3. HRP-conjugated secondary antibodies (Vectastain Elite ABC HRP Kits) were applied for 30 minutes and visualized with DAB (Vector Laboratories; SK-4100), or secondary Alexa Fluor 488 or 594 dye-conjugated antibodies (Life Technologies) applied for 1 hour at room temperature. Fluorescence antibody-labeled slides were mounted with Prolong Gold Antifade mountant (Prolong Molecular Probes; P36934) after counterstaining with DAPI.

In vitro cell assays

For knockdown studies, two independent MiRE-based shRNAs targeting *cGas* (shcGas1: CGAAGAAGTTAAAGAAATCAAA, shcGas2: CTCGAAGAAAATTGAATATGAA) were cloned into MSCV-based vectors as described previously (*8*). An shRNA targeting *Renilla* was used as a

control (*9*). Following transduction with shRNA retroviral constructs, cell selection was performed with 4 μg/mL puromycin for 3 days. Knockdown efficiency was evaluated by RT-qPCR.

For cell viability assays, two thousand cells were plated in 100 μl of media per well of a black-walled 96-well plate (Perkin Elmer). The next day, media was changed, and cells were treated with drugs for 72 hours. Following treatment, cell viability was assessed using the CellTiter-Glo Viability Assay (Promega) according to the manufacturer's protocol. IC_{50} calculations were made using Prism 6 Software (GraphPad Software). Drugs for *in vitro* studies were dissolved in DMSO. Growth medium with vehicle or drugs was changed every 3 days.

For drug withdrawal assays, cells were pretreated for 5-7 days with vehicle (DMSO) or cisplatin, and then replated (5×10^3 cells per well of 6-well plate) in the absence of drugs for 5 to 7 days. Relative growth was quantified with Crystal Violet staining.

Senescence-associated beta-galactosidase staining

Senescence-associated beta-galactosidase (SA-β-gal) staining was performed as previously described at pH 5.5 for mouse cells and tissue and pH 6 for human cells (*10*). Fresh frozen ovarian tumor sections, or adherent cells plated in 6-well plates, were fixed with 0.5% glutaraldehyde in PBS for 15 minutes, washed with PBS supplemented with 1mM $MgCl_2$, and stained for 18-24 hours in PBS containing 1 mM $MgCl_2$, 1 mg/ml X-Gal, and 5 mM each of potassium ferricyanide and potassium ferrocyanide. Tumor tissue sections were counterstained with eosin. For the fluorescent SA-β-gal labelling, frozen sections were incubated in 300 μM chloroquine solution for 30 minutes at 37ºC followed by exposure to the C12RG substrate (ImaGene Red C12RG *lacZ* Gene Expression Kit, Molecular Probes, I2906) for 2 hours at 37°C. The reaction was stopped by addition of 1 μM PETG. Slides were fixed with 4% PFA for 10 minutes at room temperature. 5 high power fields per well/section were counted and averaged to quantify the percentage of SA-β-gal$^+$ cells.

Cytokine array

EPO-GEMM derived ovarian cancer cell lines were plated in 6-well plates and treated for 48h with vehicle or cisplatin at IC50 concentrations. Conditioned media was collected, and the cells were

trypsinized and counted using a cellometer (Nexcelom Biosciences). Conditioned media samples were normalized based on cell number by diluting with complete DMEM. 50 μl aliquots of the conditioned media were analyzed using multiplex immunoassays (Mouse Cytokine/Chemokine Array 31-Plex) from Eve Technologies. Biological replicates were averaged to determine cytokine levels.

Micronuclei quantification

Cells were seeded on chamber slides. Following vehicle or cisplatin treatment, the cells were washed and fixed in 4% formaldehyde/PBS for 10 minutes at room temperature. For confocal microscopy, cells were mounted on coverslips using ProLong Gold antifade reagent with DAPI counterstaining (#P36935, Life Technologies). Images were analyzed using ImageJ/Fiji software.

Rad51 assay

Cells were irradiated with a 10 Gy dose of ionizing radiation (IR) and allowed to recover for 4 hr. Cells were fixed with 4% solution of formaldehyde in PBS for 30 min and permeabilized in 0.2% Triton X-100 in PBS^{++} (PBS solution containing 1 mM $CaCl_2$ and 0.5 mM $MgCl_2$) for 20 min. For blocking, cells were incubated for 30 min in staining buffer (1% BSA, 0.15% glycine and 0.1% Triton X-100 in PBS^{++}). Cells were incubated with primary RAD51 antibody (70-001, BioAcademia, 1:5000) in staining buffer for 2h at room temperature followed by incubation of fluorophore-conjugated secondary antibody for 1h at room temperature. Samples were mounted with Prolong Gold Antifade mountant (Prolong Molecular Probes; P36934) after counterstaining with DAPI. RAD51 foci were quantified with ImageJ/Fiji software.

Flow Cytometry

To prepare single cell suspensions for flow cytometry analysis, tumors were minced with a razorblade into small pieces and placed in 5 ml of pre-warmed collagenase buffer (1x HBSS with calcium and magnesium (GIBCO), 2 mg/ml Collagenase D (11088858001; Sigma), 0.1 mg/ml DNase I (DN25; Sigma)). Samples were then transferred to C tubes and processed using program

37C_m_TDK1_1 on a gentleMACSC Octo dissociator with heater (Miltenyi Biotec). Dissociated tissue was filtered through a 70 μm cell strainer and centrifuged at 1500 rpm for 5 minutes. Samples were resuspended in FACS buffer (1x PBS, 2% FBS, 2 mM EDTA) and 3 x 10^6 cells were seeded in a U-bottom 96-well plate. Samples were blocked with anti-CD16/32 (FC block, BD Pharmigen) for 10 minutes and then incubated with antibodies for 30 minutes on ice. In each experiment, a myeloid and a lymphoid panel were set up. The antibodies used for flow cytometry are provided in Table S4 and S5. Gates were set using fluorescence minus one (FMO) controls. Flow cytometry was performed on a LSR Fortessa or LSR II flow cytometer, and data were analyzed using FlowJo (TreeStar).

RT-qPCR

Total RNA was extracted from cell lines treated with vehicle or cisplatin for 48h using the RNeasy Mini Kit (QIAGEN). Complementary DNA (cDNA) was obtained using the TaqMan reverse transcription reagents (Applied Biosystems). Real-time quantitative PCR was performed in duplicate or triplicate using SYBR Green PCR Master Mix (Applied Biosystems) on the ViiA 7 Real-Time PCR System (Invitrogen). Expression was calculated using the $\Delta\Delta$Ct method, *Gapdh* served as an endogenous normalization control. Table S6 indicates the primer sequences used for RT-qPCR.

Figure Preparation

Figures were prepared using BioRender.com for scientific illustrations and Illustrator CC 2020 (Adobe).

Supplementary Table S1: Overview of electroporation plasmid mixes

Mouse Strain	***Plasmid Mix***	***Genotype***
WT C57BL/6	20 µg sg*Trp53*/sg*Pten* Cas9 pX330 vector<br>20 µg sg*Rb* Cas9 pX330 vector	PPtRb
WT C57BL/6	20 µg sg*Trp53/Pten* Cas9 pX330 vector	PPt
WT C57BL/6	20 µg sg*Trp53* Cas9 pX330 vector	p53_only
WT C57BL/6	1 µg SB13 transposase<br>5 µg *MYC* transposon vector<br>20 µg sg*Trp53* Cas9 pX330 vector	MP
WT C57BL/6	1 µg SB13 transposase<br>5 µg *MYC* transposon vector<br>20 µg sg*Trp53*/*Brca1* Cas9 pX330 vector	MPB1
CK8-CreER;LSL-Cas9-IRES-GFP	1 µg SB13<br>5 µg *MYC* transposon vector<br>20 µg sg*Trp53* vector	CK8-MP

Supplementary Table S2: sgRNA sequences

Gene	***sgRNA sequence***
p53	ACCCTGTCACCGAGACCCC
Pten	GTTTGTGGTCTGCCAGCTAA
Rb	TGCGCGGGGTCGTCCTCCCG
Brca1_1<br>Brca1_2	TGTTATCCAAGGAACATCGG<br>GCAGCAGGAAATGGCTCACC

Supplementary Table S3: Primary antibodies for IHC and IF

Antigen	*Manufacturer and catalogue number*
MYC	Abcam AB32072
Wilms-Tumor 1	Abcam AB89901
Cytokeratin-7	Abcam AB181598
Granzyme B	Abcam AB4059
Ki67	Abcam AB16667
Cancer antigen 125	Abbiotec 250566
Pax8	Proteintech 10336
CD8	Ebioscience 4SM15
Cleaved Caspase3	Cell Signaling 9664
γH2AX	Millipore JBW301
53BP1	Novus Biologicals NB100-305

Supplementary Table S4: Antibodies used for flow cytometry analysis (myeloid panel)

Antigen	*Fluorophore*	*Company*	*Clone #*	*Catalogue #*
CD45	AF700	Biolegend	30-F11	103128
CD3	BUV737	BD	17A2	612803
Ly6G	BV605	BD	1A8	563005
SIGLECF	PerCp-Cy5.5	BD	E50-2440	565526
LY6C	APC-C7	Biolegend	HK1.4	128026
CD11b	BUV395	BD	M1/70	563553
CD11c	BV785	Biolegend	N418	117335
MHCII	AF488	Biolegend	M5/114.15.2	107616
F4/80	PE-eFluor610	ThermoFisher	BM8	61-4801-82
CD19	BV650	BD	1D3	563235
CD103	PE	Biolegend	2E7	121405
PD-L1	APC	Biolegend	10F.9G2	124312
CD80	BV421	Biolegend	16-10A1	104725
CD206	BV711	Biolegend	C068C2	141727
Viability	eFluor506	ThermoFisher	-	65-0866-18

Supplementary Table S5: Antibodies used for flow cytometry analysis (lymphoid panel)

Antigen	*Fluorophore*	*Company*	*Clone #*	*Catalogue #*
CD45	AF700	Biolegend	30-F11	103128
CD3	AF488	Biolegend	17A2	100210
CD4	BUV395	BD	GK1.5	563790
CD8	PECy7	Biolegend	53-6.7	100722
CD25	BV605	Biolegend	PC61	102035
CD69	Percp-Cy5.5	Biolegend	H1.2F3	104522
CD62L	BV421	BD	MEL-14	562910
CD44	ApC-Cy7	BD	IM7	560568
PD1	PE	Biolegend	29F.1A12	135206
NK1.1	APC	Biolegend	PK136	108710
TIM3	BV711	Biolegend	RMT3-23	119727
LAG3	BV650	Biolegend	C9B7W	125227
KLRG1	BV785	Biolegend	2F1	138429
Viability	eFluor506	ThermoFisher	-	65-0866-18

Supplementary Table S6: Primer sequences used for RT-qPCR

cGas_Fw	GAGGCGCGGAAAGTCGTAA
cGas_Rv	TTGTCCGGTTCCTTCCTGGA
Ccl5_Fw	ATATGGCTCGGACACCACTC
Ccl5_Rv	TCCTTCGAGTGACAAACACG
Cxcl10_Fw	CCCACGTGTTGAGATCATTG
Cxcl10_Rv	GTGTGTGCGTGGCTTCACT
IL6_Fw	ACCAGAGGAAATTTTCAATAGGC
IL6_Rv	TGATGCACTTGCAGAAAACA

Supplementary References

1. A. Jiménez-Sanchez *et al.*, Unraveling tumor–immune heterogeneity in advanced ovarian cancer uncovers immunogenic effect of chemotherapy. *Nature Genetics*, 1–21 (2020).

2. A. Van Keymeulen *et al.*, Distinct stem cells contribute to mammary gland development and maintenance. *Nature*. **479**, 189–193 (2011).

3. A. M. Bolger, M. Lohse, B. Usadel, Trimmomatic: a flexible trimmer for Illumina sequence data. *Bioinformatics*. **30**, 2114–2120 (2014).

4. A. Dobin *et al.*, STAR: ultrafast universal RNA-seq aligner. *Bioinformatics*. **29**, 15–21 (2012).

5. S. Anders, P. T. Pyl, W. Huber, HTSeq--a Python framework to work with high-throughput sequencing data. - PubMed - NCBI. *Bioinformatics*. **31**, 166–169 (2015).

6. M. I. Love, W. Huber, S. Anders, Moderated estimation of fold change and dispersion for RNA-seq data with DESeq2. *Genome Biol.* **15**, 1–21 (2014).

7. O. M. T. Pearce *et al.*, Deconstruction of a Metastatic Tumor Microenvironment Reveals a Common Matrix Response in Human Cancers. *Cancer Discovery*. **8**, 304–319 (2018).

8. A. Chicas *et al.*, Dissecting the Unique Role of the Retinoblastoma Tumor Suppressor during Cellular Senescence. *Cancer Cell*. **17**, 376–387 (2010).

9. M. Saborowski *et al.*, A modular and flexible ESC-based mouse model of pancreatic cancer. *Genes Dev.* **28**, 85–97 (2014).

10. V. Krizhanovsky *et al.*, Senescence of activated stellate cells limits liver fibrosis. *Cell*. **134**, 657–667 (2008).
